# A revision of the genus *Pseudoechthistatus* Pic (Coleoptera, Cerambycidae, Lamiinae, Lamiini)

**DOI:** 10.3897/zookeys.604.9049

**Published:** 2016-07-11

**Authors:** Wen-Xuan Bi, Mei-Ying Lin

**Affiliations:** 1 Key Laboratory of Zoological Systematics and Evolution, Institute of Zoology, Chinese Academy of Sciences, Beichen West Road, Chaoyang, Beijing, 100101, China; 2 Room 401, No. 2, Lane 155, Lianhua South Road, Shanghai, 201100, China

**Keywords:** Taxonomy, new species, Lamiini, endophallus, China, Vietnam, Myanmar, Oriental region

## Abstract

The genus *Pseudoechthistatus* Pic, 1917 is redefined and revised. Five species of the genus are described as new, *Pseudoechthistatus sinicus* **sp. n.** and *Pseudoechthistatus chiangshunani* **sp. n.** from central Yunnan, China, *Pseudoechthistatus pufujiae* sp. n. from western Yunnan, China, and *Pseudoechthistatus holzschuhi* **sp. n.** and *Pseudoechthistatus glabripennis* **sp. n.** from southern Yunnan and northern Vietnam. *Pseudoechthistatus birmanicus* Breuning, 1942 is excluded from the fauna of China. Three poorly known species, *Pseudoechthistatus obliquefasciatus* Pic, 1917, *Pseudoechthistatus granulatus* Breuning, 1942, and *Pseudoechthistatus acutipennis* Chiang, 1981 are redescribed, and the type localities of the former two species are discussed. Endophallic structure of seven species in inflated and everted condition are studied and compared with their relatives. Illustrations of habitus and major diagnostic features of all species are provided. Some biological notes are reported. An identification key as well as a distributional map are presented.

## Introduction

The little-known genus *Pseudoechthistatus* Pic, 1917 was established based on a flightless species, *Pseudoechthistatus obliquefasciatus* Pic, 1917 from Dali, Yunnan, China. Later, Breuning (1942) revised the genus in his revision of the Phrissomini and added two species, *Pseudoechthistatus birmanicus* from Myanmar and *Pseudoechthistatus granulatus* from Tatsienlou (Kangding), Sichuan, China. Chiang (1981) described *Pseudoechthistatus acutipennis* from Mt. Omei (Emeishan), Sichuan, China as the fourth species of the genus. Hence, in the Titan database (Tavakilian and Chevillotte 2015), a total of four valid species was included in *Pseudoechthistatus*.

Specimens of the genus *Pseudoechthistatus* were so rare that all four species were described from single specimens and only a few additional specimens have been reported since the original publications. Li (1988) listed “Fugong, Yunnan” as an additional locality for *Pseudoechthistatus granulatus* (voucher specimen not available to the authors). This datum referred to Pu (1992). In the same paper, Pu (1992) reported *Pseudoechthistatus birmanicus* from Yaojiaping, Yunnan as a new country record for China based on a single female specimen. Ultimately for Chinese fauna, three species were included in Hua (2002) and four species were listed by Löbl and Smetana (2010) in their catalogues.

In the course of our studies of material from several major collections and from several expeditions to Yunnan, China, during 2010 to 2015, five new species were discovered (including four winged species). The generic definition of *Pseudoechthistatus* is broadened to legitimately include all those species. The four known species were determined based on high-quality photographs of their type specimens, three of them were reexamined and redescribed based on fresh material. *Pseudoechthistatus birmanicus* is excluded from the fauna of China, and the type localities of *Pseudoechthistatus obliquefasciatus* and *Pseudoechthistatus granulatus* are discussed. Endophallic structure of seven species in inflated and everted condition are described, figured and compared with their relatives from *Paraleprodera* Breuning, 1935. The basic observing method for endophallic comparison is discussed and proposed to be done in everted and inflated condition at least in Lamiini *sensu lato*.

Specimens are deposited in the following institutions, museums or private collections; abbreviations as shown in the text:

CBWX
 Collection of Wen-Xuan Bi, Shanghai, China 


CCCC
 Collection of Chang-Chin Chen, Tianjin, China 


CCH
 Collection of Carolus Holzschuh, Villach, Austria 


CGQH
 Collection of Gui-Qiang Huang, Chongqing, China 


CHTL
 Collection of Tian-Long He, Huainan, Anhui, China 


CJM
 Collection of Ming Jin, Shanghai, China 


CLB
 Collection of Bin Liu, Beijing, China 


CLC
 Collection of Chao Li, Beijing, China 


CSXB
 Collection of Xiao-Bin Song, Shanghai, China 


CTT
 Collection of Tomáš Tichý, Opava, Czech Republic 


CZDY
 Collection of De-Yao Zhou, Shanghai, China 


IZAS
Institute of Zoology, Chinese Academy of Sciences, Beijing, China 


MHBU
Museum of Hebei University, Baoding, China 


MNHN
Muséum National d’Histoire Naturelle, Paris, France 


NHMB
 Naturhistorisches Museum (Museum Frey, Tutzing), Basel, Switzerland 


NHRS
 Naturhistoriska riksmuseet, Stockholm, Sweden 


NMNH
National Museum of Natural History (Smithsonian Institution), Washington, USA 


SHEM
 Shanghai Entomology Museum, Chinese Academy of Sciences, Shanghai, China 


SWU
 College of Plant Protection, Southwest University, Chongqing, China 


Labels of the type specimens are quoted verbatim; double quotation marks (“ ”) are used for a single label, a slash (/) is used to separate lines on the same label, italics indicate handwriting, notes are included in [], Chinese characters are transcribed in the modern system.

Terminology of endophallic structures follows Danilevsky et al. (2005), Danilevsky and Kasatkin (2006) and Yamasako and Ohbayashi (2011). The abbreviations used in the present paper are as follows: APH – apical phallomere; BPH – basal phallomere; CS – crescent shaped sclerites; CT – central trunk; MPH – median phallomere; MT – medial tube; PB – preapical bulb; ab – apical bulb; af – apical furrow; bb – apical bubble; bs – basal swelling of central trunk; gn – gonopores; im – internal membrane of apical furrow; vs – ventral swelling of central bladder; ltc – lateral tubercles of central trunk, vbt – ventral basal tubercle of preapical bulb (first-time used herein).

## Taxonomy

### Pseudoechthistatus

Taxon classificationAnimaliaColeopteraCerambycidae

Pic, 1917

Pseudoechthistatus Pic, 1917: 6. Type species: Pseudoechthistatus obliquefasciatus Pic, 1917, by monotypy.
Pseudechthistatus (sic): Breuning 1942: 132; Breuning 1961: 318; Löbl and Smetana 2010: 286.
Pseudoechthistatus : Gressitt 1951: 349; Chiang et al. 1985: 104.

#### Redescription.

Body elongate, medium sized (ca. 15.0–25.0 mm long). Head subequal to the pronotal width at base. Eyes coarsely faceted, strongly emarginate; lower lobe small, weakly prominent, subequal to or slightly longer than width. Frons wider than long. Antennal tubercles moderately prominent and separated. Antennae long, ca. 1.6–2.0 times (in male) or 1.2–1.4 times (in female) as long as body length; scape moderately long, apical cicatrix completed, the 3^rd^ antennomere longest, ca 1.5–1.8 times as long as scape, 4^th^ antennomere slightly longer than (in male) or subequal to (in female) scape, 4^th^ to 10^th^ successively shortened and narrowed, last antennomere slightly longer than penultimate; basal antennomeres (3–4 in male, 4–7 in female) distinctly fringed beneath. Both maxillary and labial terminal palpomeres fusiform. Pronotum cylindrical, subequal to or slightly longer than width at base; with two indistinct transverse grooves at the anterior and posterior margins; disk with a rugose longitudinal ridge, slightly raised medially, both sides with a developed or reduced, longitudinal pubescent band; with a lateral spine moderate long and acute apically at anterior middle of each side; prosternal intercoxal process narrow, slightly emarginate at apex, lower than coxae; procoxal cavities closed posteriorly; mesosternal intercoxal process without tubercle and obliquely sloped in lateral view; mesocoxal cavities open externally to mesepimera; metasternum short to moderately long, ca 1.0–1.8 times as long as mesosternal length. Scutellum broadly rounded posteriorly. Elytra elongate, ca. 1.8–2.2 times as long as humeral width, widest at the middle or at humeri or subparallel-sided in basal half, gradually to strongly narrowed after the middle, rounded or obliquely truncated to acute apically; disk finely to coarsely punctured, granules absent or moderately to strongly developed, with few erect or suberect setae; each elytron conspicuously with a moderate to large sized, median, moderately to strongly raised, glabrous tubercle subbasally (Figs 38–40); generally provided with three light pubescent markings: the first annular marking around the subbasal tubercle (subbasal annular marking), the second band complete or discontinuous, nearly transverse to strongly oblique, across the elytron near middle (middle band), the third stripe longitudinally near apical one-fourth toward elytral apex (preapical stripe). Hindwings developed to highly reduced. Legs long and slender, protibia with a subapical tooth beneath (weak in females), mesotibia with a subapical oblique groove externally, tarsus four segmented. Tarsal claws free, divaricate to moderately divergent.

**Male genitalia.** Tergite VIII (Figs 49–55, a) trapezoidal, truncated to slightly emarginated apically, with moderately long setae. Tegmen (Figs 49–55, b, c) in lateral view moderately curved, rhombic in shape and widest at middle or behind middle in ventral view; lateral lobes moderately slender, ca. one-fourth of total length of tegmen, which moderately provided with long setae on apex. Median lobe (Figs 49–55, d, e) slightly shorter than tegmen; moderately curved in lateral view; apex rounded to subacuminate in antero-dorsal view. Endophallus in everted condition (Figs 58–64) S-shaped, long and slender, subequal to or slightly longer than triple length of median lobe; BPH, MPH and APH well defined, crescent shaped sclerites (cs) present, MPH subdivided into MT, CT and PB by a constriction; the length of MT ca. 2.0–2.5 times as long as the length of BPH, CT slightly shorter than PB, the combined length of CT and PB subequal to the length of BPH and slightly shorter than median lobe; BPH hardly swollen apically; PB cylindrical at base with developed anterior bulb, CT developed, basal swelling (bs) strongly swollen ventrolaterally or with distinct lateral tubercles (ltc), slightly swollen posterodorsally, MT with ventral swelling (vs) generally rudimentary; APH variable, moderately to strongly constrictive or moderately swollen, apical bulb (ab) sclerotized apically or at least in ventral side (when APH strongly constrictive), apical furrow (af) with internal membrane (im) well developed (Fig. 58b); apical bulb (ab), apical part of CT and PB, ventral side of basal swelling (bs) provided with spicules; ejaculatory ducts paired; gonopores (gn) situated near apex of apical bubble (bb), a pair of rod-like sclerite generally absent.

**Female genitalia.** Setae of sternites VIII sparse and short, apical ones longer (Fig. 56f). Distinct lateral notch present and positioned behind the distinct pigmented patch on sternites VIII (Fig. 56f). The paraproct is very short and devoid of baculi; the coxite lobes are very long and bear small styli (Figs 56g, 56h). Spiculum ventrale (Fig. 56f) longer than abdomen, slightly expanded apically. Female genital track (Fig. 57) with well-developed vaginal plate (VP); bursa copulatrix (BC) moderately long, spermathecal duct attached before middle of bursa, compose of a thin long duct and an expanded and curved apical part. Spermathecal capsule (SP) and gland (SPGL) positioned on apex of spermathecal duct (SPD). Spermathecal capsule strongly sclerotised, crutch shaped (Fig. 57a), apical part more than twice of basal part in length, the whole capsule larger than the expanded apical part of spermathecal duct. Spermathecal gland is an elongate membranous sac, with its length variable but always more than triple length of spemathecal duct.

#### Distribution

**Map 1.**
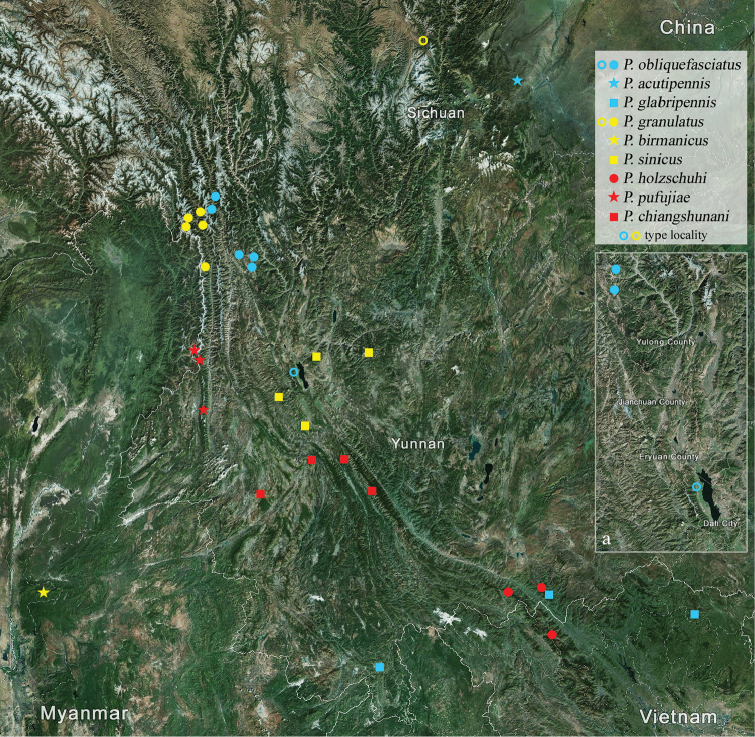
Distribution of the species of *Pseudoechthistatus*. **a** enlargement of Dali area.

(Map 1). China, Myanmar, Vietnam.

#### Remarks.

This genus is unique with a conspicuous raised subbasal tubercle on each elytron among the oriental genera of Lamiini. It is superficially resembles *Mesechthistatus* Breuning, 1950, but immediately distinguished by antennal scape with a complete cicatrix, basal antennomeres distinctly fringed beneath, pronotum with a rugose median longitudinal ridge, and elytra lacking lateral carinae. *Pseudoechthistatus* shares some characters with *Paraleprodera* Breuning, 1935: antennae normal (without swollen), scape with a complete cicatrix, basal antennomeres distinctly fringed beneath, pronotal lateral spine present, prosternal process normal (not angularly enlarged between coxae), protibia with a subapical tooth beneath (at least in male), and similar to some species of *Paraleprodera* (e.g. *Paraleprodera diophthalma*, *Paraleprodera bisignata*, *Paraleprodera bigemmata*) by possess the subbasal tubercle (or tubercles) on each elytron, but is distinguished by elytron with single large raised subbasal tubercle, pronotum with a rugose longitudinal ridge medially, endophallus with CT developed, swollen in dorsal and ventral sides and APH without a pair of U-shaped sclerite (the latter with the subbasal tubercle composed of small granules, CT of endophallus simple and APH with a pair of U-shaped sclerite (Figs 67f, 68g)). It is most close to another group of *Paraleprodera* (e.g. *Paraleprodera carolina*, *Paraleprodera itzingeri*, *Paraleprodera mesophthalma*) with regard to the overall form, especially the presence of the median rugose longitudinal ridge on pronotum, the shape and proportion of the endophallus and the absence of the U-shaped sclerite on APH. But it is distinguished from them by elytron with a subbasal tubercle, endophallus with CT swollen postero-dorsally and PB cylindrical at base (without a ventral tubercle (vbt) (Figs 65, 66)).

Breuning (1942) mentioned that *Pseudoechthistatus* has the claws “divergent” (divergence less than 90°). According to our observation, the claws of this genus are free, and most species have them “divaricate” (divergence exceeding 100°), only some species or individuals (especially of the type species) have the claws in transitional (divergence between 80° to 90°).

The subbasal tubercle on each elytron of this genus is usually single and complete. However, a few individuals (two of nearly one hundred specimens) have the subbasal tubercle separated by several grooves (Fig. 29). This was considered an aberration and is not included in the generic diagnosis, but it may indicate that the single subbasal tubercle have originated from several converging small tubercles (or granules) as present in e.g. *Paraleprodera diophthalma* Pascoe.

The type species of this genus is flightless, having a shortened metasternum (subequal to mesosternum in length), constricted humeri and reduced hindwings. These three related structures were considered as generic characters by Breuning (1942). However, *Pseudoechthistatus birmanicus* with the normal metasternum length (metasternum / mesosternum length ratio ca. 1.8) and normal hindwings, while *Pseudoechthistatus acutipennis* is transitional (metasternum / mesosternum length ratio ca. 1.5). Therefore, at least for this genus, the shortened metasternum should be treated as an infrageneric apomorphy.

*Pseudoechthistatus* was placed originally in the tribe Phrissomini by Breuning (1942), and this was followed by Gressitt (1951) and Breuning (1961). Sama (2008) synonymized Phrissomini with Lamiini. Löbl and Smetana (2010) placed the genus *Pseudoechthistatus* under the tribe Monochamini which was separately used from Lamiini. In this paper, we place it under Lamiini and follow Breuning (1943, as Agniini), Gressitt (1951), Breuning (1961) and Ohbayashi and Niisato (2007) who consider Lamiini to include Monochamini (sensu Löbl and Smetana 2010).

### Pseudoechthistatus obliquefasciatus

Taxon classificationAnimaliaColeopteraCerambycidae

Pic, 1917

Figures 1 , 2 , 17 , 21 , 29 , 30 , 38 , 41 , 49 , 56 , 57–58 , Map 1

Pseudoechthistatus obliquefasciatus Pic, 1917: 7. Type locality: Tali, Yunnan, China. Type depository: MNHN.
Pseudechthistatus (sic) obliquefasciatus: Breuning 1942: 133; Hua 2002: 227; Hua et al. 2009: 466; Löbl and Smetana 2010: 286.
Pseudoechthistatus obliquefasciatus : Gressitt 1951: 349; Chiang et al. 1987: 694; Li 1988: 46; Pu 1992: 601; Li 2009: 159.

#### Type material examined.

Holotype (Fig. 17), female, “Tali / Hte yunnan”, “*Pseudoechthistatus Pic* / *obliquefasciatus Pic* ”, “?? / ?? / *(cc? Breuning)*”, “*vari? echthistatus* / *Pascoe* / ??? / *p. 359*”, “*gene echthistatus* / *n. sp.* ? / (? *in coll Boppe*)”, “Museum Paris / Coll. M. Pic”, “*type*”, “*type*”, “TYPE” [red label] examined through five photographs taken by N. Ohbayashi in MNHN, 2014, the hand-written labels are mostly illegible.

#### Additional material examined

(6 males, 12 females): 1 female, Yunnan Weixi Pantiange, 2900 m, 1981.VII.21, leg. Shu-Yong Wang (IZAS, IOZ(E) 1904795); 1 female, Yunnan Weixi Pantiange, 2920 m, 1981.VII.22, leg. Xue-Zhong Zhang (IZAS, IOZ(E) 1904796); 1 female, CHINA, Yunnan, Weixi, Pantiange, Zhazi; N27.34904°, E99.28188°–N27.34647°, E99.27661°, 2917–3029 m, 2009.VII.10, Shi H.L. coll., beating (IZAS, IOZ(E) 1905218); 3 females, Yunnan, Diqing, Weixi, Najieluo, 2921 m, 2014.VI.29, leg. Xiao-Dong Yang (CCCC); 2 males, 1 female, ditto but 2872 m, 2014.VII.1 (CCCC); 1 male, ditto but 3106 m (CCCC); 1 female, Yunnan, Lijiang, Yulong, Ludian, 3219m, 2014.VII.3, leg. Xiao-Dong Yang (CCCC); 2 males, 3 females, Yunnan, Pass 50 km W Judian, 2005.VI.11–13, leg. Ivo Jeniš (CCH); 1 male, 1 female, Yunnan, Yanmen, 2005.VI.13–23, leg. E. Kučera (CCH).

#### Redescription.

**Figures 1–8.**
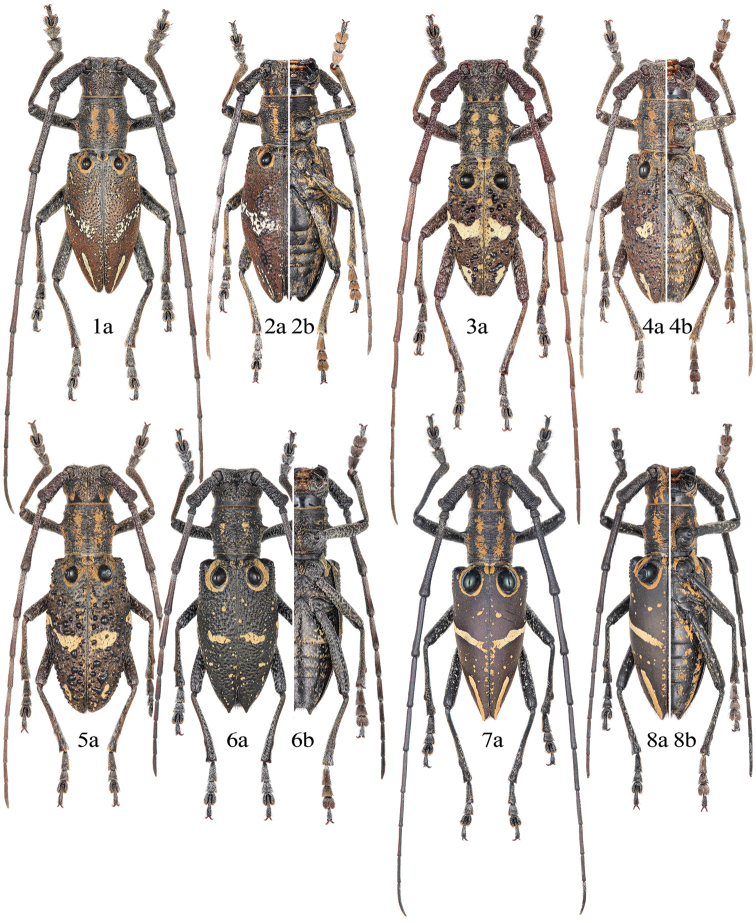
Habitus of *Pseudoechthistatus* spp. **1–2** *Pseudoechthistatus obliquefasciatus* Pic, 1917 **3–5** *Pseudoechthistatus granulatus* Breuning, 1942 **6** *Pseudoechthistatus acutipennis* Chiang, 1981 **7–8** *Pseudoechthistatus glabripennis* sp. n. (paratype) **1, 3, 7** male **2, 4–6, 8** female. **a** dorsal view **b** ventral view. Not to scale.

**Male** (Fig. 1). Body length 18.0–22.2 mm, humeral width 5.5–6.2 mm. Body dark brown; head, pronotum and ventral surface covered with intermixed light yellowish and tawny pubescence. Head with four short tawny vittae behind upper eye lobes. Antennal scape, pedicel and 3^rd^ antennomere with sparse pale pubescence, 4^th^ to 9^th^ antennomeres with same color of pubescence at basal half. Pronotum with two longitudinal tawny bands on each side of disk and other two longitudinal bands on lateral margins postmedially, the discal bands distinctly longer than half of pronotal length. Scutellum densely clothed with tawny pubescence, sparse along middle. Elytron with pubescence predominantly brown, with tawny pubescence forming the subbasal annular marking and few small spots scattered at basal one fifth, with light yellowish pubescence forming the middle band and the preapical stripe; the middle band moderately broad, strongly oblique (inclined at an angle of 40 to 50 degrees to the transverse axis), complete or interrupted or dispersed into small spots, hardly reaching suture; the preapical stripe narrow, well developed, subequal to or slightly shorter than one-fourth of elytral length. Legs (Fig. 30) clothed with yellowish and tawny pubescence of which the tawny one forming small spots moderately scattered on femora and becoming denser on tibiae.

Body elongate, oblong oval. Head (Fig. 21) with frons moderately punctured; lower eye lobe subequal in length and width, 0.7 times as long as gena. Antennae 1.9 times as long as body length, surpassing elytral apex by six antennomeres; 3^rd^ antennomere ca. 1.8 times as long as scape, ca. 1.2 times as long as 4^th^ antennomere; scape and 3^rd^ antennomere coarsely punctured; scape to 3^rd^ or 4^th^ antennomere sparsely fringed beneath. Elytra ca. 1.4 times as wide as pronotal base, 2.0–2.1 times as long as humeral width; humeri slightly constricted, widened at basal two-fifth, then convergent toward rounded apices; disk moderately punctured, slightly denser near suture, becoming shallower at apical one-third, moderately granulated on basal half, becoming weaker anteriorly; subbasal tubercle moderate in size, as wide as or slightly narrower than scutellar width. Hindwings (Fig. 41) strongly reduced, 0.7 times as long as elytral length. Legs long and slender, metatibiae exceeding elytral apices at base.

**Male genitalia** (Figs 49, 58). Tergite VIII (Fig. 49a) transverse, slightly emarginated apically and nearly straight at sides, length 0.9 times as long as width. Tegmen (Fig. 49b–c) with lateral lobe widest at base, gently narrowed at basal one-third, then slightly dilated toward rounded apex. Median lobe (Fig. 49d–e) with apex subacute in antero-dorsal view. Endophallus (n = 3, Fig. 58) subequal to triple length of median lobe, the length of MT ca. 2.4 times as long as the length of BPH, the length of CT+PB slightly longer than the length of BPH; MPH strongly curved at apical one-third, PB cylindrical at basal one-third, basal swelling (bs) of CT moderately developed; APH moderately constrictive, ca. 0.6 times as wide as the maximum width of PB at base, with apical bulb (ab) heavily sclerotized apically in ventral side (Fig. 58c), obliquely truncated in lateral view; small spicules sparsely distributed on apical bulb and anterior margin of PB.

**Female** (Fig. 2). Body length 16.0–22.1 mm, humeral width 4.6–6.4 mm. Almost identical to male in general appearance. Antennae ca. 1.2 times as long as body length, apical three antennomeres surpassing elytral apex; scape to 5^th^ or 6^th^ antennomere fringed beneath; pronotum subequal in length and basal width; elytron longer in proportion to body length (elytra 2.2–2.3 times as long as humeral width); legs comparatively short, metatibiae exceeding elytral apices at basal half. Female genitalia as Fig. 56.

#### Diagnosis.

Elytron with predominant brown pubescence, middle band strongly oblique, subbasal tubercle as wide as or slightly narrower than scutellar width; elytral apices rounded; humeri slightly constricted; hindwings strongly reduced. Endophallus with APH constrictive, apical bulb (ab) heavily sclerotized apically in ventral side, obliquely truncated in lateral view.

#### Distribution

(Map 1). China: Yunnan.

#### Remarks.

Slightly intraspecific variation can be observed between the population from northern area (Weixi County: Najieluo; Deqin County: Yanmen) and southern area (Weixi County: Pantiange; Yulong County: Ludian, Judian), the northern population with elytra relatively long: elytral length / humeral width ca. 2.1 in male and 2.3 in female, while in southern population, elytral length / humeral width ca 2.0 in male and 2.2 in female. And the holotype is identical to the population from southern area. Currently, sympatry has not been confirmed among the flightless species of this genus, with the exception that the holotype from “Tali” seemingly overlaps with the range of another flightless species, *Pseudoechthistatus sinicus*. However, the old name “Tali” or “Tali Fu” (Breuning, 1942), which had been abandoned in 1913 covered a vast region including today’s Dali City, Yunlong County, Eryuan County, Binchuan County, Xiangyun County etc. (Dai et al. 2005: 164). The exact type locality of this species is difficult to determine unless further information is acquired. However, based on the similarity of external characters, we conclude that the type specimen might have been collected from the north of Eryuan county or further north (Map 1a).

### Pseudoechthistatus acutipennis

Taxon classificationAnimaliaColeopteraCerambycidae

Chiang, 1981

Figures 6 , 20 , 28 , 32 , 44 , Map 1

Pseudoechthistatus acutipennis Chiang, 1981: 80, 84, pl. 1, fig. 7. Type locality: Mt. Omei, Sichuan, China. Type depository: SWU.
Pseudoechthistatus acutipennis : Chiang et al. 1985: 104, pl. VII, fig. 111.
Pseudechthistatus (sic) acutipennis: Hua 2002: 227; Hua et al. 2009: 246, 390, pl. CIX, 1252; Löbl and Smetana 2010: 286.

#### Type material examined.

Holotype (Fig. 20), female, “Sichuan Emeishan *Jiulaodong* / 196*2.VII.9* / *Chen Li-Juan et al.*”, “*Pseudechthistatus* / *acutipennis* *sp. n.* / det. Chiang Shu-Nan 19*78*”, “Holotype” [red label] examined through two photographs provided by Li Chen from SWU, 2014.

#### Additional material examined.

1 female, Sichuan, Emeishan, Jiulinggang, 1900 m, 2014.VIII.7, leg. De-Yao Zhou (CZDY).

#### Redescription.

**Female** (Fig. 6). Body length 17.0–18.0 mm, humeral width 5.2–5.4 mm. Body brownish black; head, pronotum sparsely covered with pale and tawny pubescence, ventral surface with intermixed pale and grayish yellow pubescence forming small spots scattered throughout. Antennal scape, pedicel and basal half of 3^rd^ antennomere with sparse pale pubescence, 4^th^ to 8^th^ antennomeres indistinctly with the same pubescence at base, remainder with fine brown pubescence. Pronotum with a pair of longitudinal tawny bands on each side of disk, slightly shorter than one-third of pronotal length. Scutellum clothed with tawny pubescence, except a median glabrous line. Elytron with tawny pubescence forming the subbasal annular marking and some small spots sparsely scattered throughout; with the same pubescence forming the middle band, which moderately oblique, widely interrupted near lateral margin, transversely near suture; remainder with very fine dark brown pubescence. Legs (Fig. 32) moderately clothed with intermixed pale and yellowish pubescence interrupted by scattered glabrous spots.

Body elongate, oblong oval. Head (Fig. 28) with frons densely and coarsely punctured; lower eye lobe 1.2 times as long as width, 0.8 times as long as gena. Antennae 1.2 times as long as body length, surpassing elytral apex by three antennomeres; 3^rd^ antennomere 1.5 times as long as scape, ca. 1.4 times as long as 4^th^ antennomere; scape coarsely punctured; scape to 4^th^ antennomere sparsely fringed beneath. Pronotum subequal in length and basal width, lateral spine short, slightly thickened at base, with acute apex; metasternum 1.5 times as long as mesosternal length. Elytra ca. 1.5 times as wide as pronotal base, 2.1 times as long as humeral width; subparallel-sided in basal one-third, very weakly widened at middle, then moderately convergent toward strongly acute apices; disk densely and coarsely punctured, moderately granulated on basal half and near humerus; subbasal tubercle moderately developed and raised, ca. 1.2 times as wide as scutellar width. Hindwings (Fig. 44) reduced, 1.3 times as long as elytral length. Legs moderately long and slender, metatibiae exceeding elytral apices at basal one-third.

**Male.** Unknown.

#### Diagnosis.

Body and elytra brownish black, very finely pubescent (besides the tawny pubescent markings); pronotal longitudinal bands reduced, shorter than one-third of pronotal length; elytral middle bands widely interrupted near lateral margin, preapical stripe absent; elytral apices strongly acute, disk densely and coarsely punctured; hindwings reduced.

#### Distribution

(Map 1). China: Sichuan.

### Pseudoechthistatus birmanicus

Taxon classificationAnimaliaColeopteraCerambycidae

Breuning, 1942

Figure 18 , Map 1

Pseudechthistatus (sic) birmanicus Breuning, 1942: 133. Type locality: Ruby Mines, Myanmar. Type depository: NHMB.
Pseudoechthistatus birmanicus : Pu 1992: 601 [misidentification].
Pseudechthistatus (sic) birmanicus: Hua et al. 2009: 465; Löbl and Smetana 2010: 286 [partly identified].

#### Type material examined.

Holotype (Fig. 18), male, “H^te^ Birmanie / Mines des Rubis / 1200 m–2300 m / Doherty 1890”, “*Pseudechthistatus* / *birmanicus* / *mihi Type*! / det. Breuning” examined through three photographs taken by J. Yamasako and N. Ohbayashi in NHMB, 2012.

#### Redescription

(based on quality photographs, and modified from the original description). **Male.** Body length 21.0 mm, body width 7.5 mm. Body dark brown, body covered with tawny and brown pubescence. Head with four short tawny vittae behind upper eye lobes. Antennal scape with sparse light yellowish pubescence, basal half of 3^rd^ antennomere with sparse fine light yellowish pubescence. Pronotum with paired discal longitudinal band rather long, longer than two-thirds of pronotal length. Elytron with pubescence predominantly brick-red; middle pubescent band light yellowish, broad, well defined, nearly transverse, reaching suture; the preapical stripe same color as middle band, well developed, moderately broader at base. Body elongate. Antennae 1.7 times as long as body length, surpassing elytral apex by five antennomeres; 3^rd^ antennomere 1.7 times as long as scape, 1.1 times as long as 4^th^ antennomere; scape moderately punctured, 3^rd^ antennomere sparsely punctured on basal half; scape to 3^rd^ antennomere fringed beneath. Pronotum slightly longer than width at base, lateral spine short, slightly thickened at base with moderate acute apex; metasternum 1.8 times as long as mesosternal length. Elytra 1.6 times as wide as pronotal base at humeri, 1.8 times as long as humeral width; subparallel-sided in basal one-fourth, very weakly widened a little before middle, then moderately convergent toward subacute apices; disk sparsely and finely punctured, sparsely provided with large but flat granules extending to apical one-fourth; subbasal tubercle close to elytral base, moderately developed and raised, ca. 1.3 times as wide as scutellar width. Hindwings developed, distinctly longer than elytral length.

#### Distribution

(Map 1). Myanmar: Mandalay (Mogok = Ruby Mines).

#### Remarks.

This species is only known from its type locality, Ruby Mines (= Mogok), Myanmar at present. Based on our examination of photos of the holotype, the distribution of this species in Yunnan, reported by Pu (1992) is considered a misidentification of *Pseudoechthistatus pufujiae* sp. n., which is described in this paper.

### Pseudoechthistatus granulatus

Taxon classificationAnimaliaColeopteraCerambycidae

Breuning, 1942

Figures 3–5 , 19 , 22 , 31 , 39 , 42 , 50 , 60 , 69 , 70 , Map 1

Pseudechthistatus (sic) granulatus Breuning, 1942: 133. Type locality: Tatsienlu (?). Type depository: NHMB
Pseudoechthistatus granulatus : Gressitt 1951: 349; Li 1988: 46; Pu 1992: 601; Li 2009: 158, 182.
Pseudechthistatus (sic) granulatus: Hua 2002: 227; Hua et al. 2009: 465; Löbl and Smetana 2010: 286.

#### Type material examined.

Holotype (Fig. 19), female, “*Tatsienlu*”, “*Pseudechthistatus* / *granulatus* / *mihi Type*! / det. Breuning” examined through three photographs taken by J. Yamasako & N. Ohbayashi in NHMB, 2012.

#### Additional material examined.

(22 males, 20 females): 1 male, Yunnan Prov., Gaolinggongshan, Fugong County, Shiyueliangxiang, Shibaliyingdi, 3105 m, 27.18380°N, 98.71021°E, 2004.V.7 night, leg. Hong-Bin Liang (IZAS, IOZ(E) 1904798); 1 male, 1 female, Yunnan, Fugong, Shibaliyingdi, 3105 m, 2005.VIII.9, leg. Hong-Bin Liang (CBWX); 1 male, CHINA, Yunnan Prov. Gongshan County, No12 Bridge–Yakou, 2750–3680 m, N27.43, E98.28, 2000.VII.18, leg. H. B. Liang, Sino-America Exped. (IZAS, IOZ(E) 1904797); 2 females, Yunnan, Gongshan, Gabocun, 2478 m, 2014.VI.14, leg. Xiao-Dong Yang (CCCC); 2 females, ditto except 2500 m, 2015.VI.16, leg. Wen-Xuan Bi (CBWX); 1 male, Yunnan, Gongshan, Sendang–Dabadi, 2834 m, 2014.VI.16, leg. Xiao-Dong Yang (CCCC); 1 male, 1 female, ditto except 2840 m, 2015.VI.20, leg. Wen-Xuan Bi (CBWX); 1 male, ditto except leg. Yu-Tang Wang (CCCC); 1 female, ditto except leg. Xiao-Dong Yang (CCCC); 1male, ditto except Dabadi, 3020 m, 2015.VIII.11, leg. Wen-Xuan Bi (CBWX); 12 males, 6 females, Yunnan, Gongshan, Nageluo, 2850–2750 m, 2015.VI.12, leg. Wen-Xuan Bi (CBWX); 1 female, ditto except 2750 m, leg. Yu-Tang Wang (CCCC); 3 males, 4 females, ditto except 2015.VI.15, leg. Wen-Xuan Bi (CBWX); 1 female, ditto except 2750 m, leg. Chao Wu (CBWX); 1 female, ditto except 2015.VIII.12, leg. Xiao-Dong Yang (CCCC).

#### Redescription.

Male. (Fig. 3). Body length 15.0–16.8 mm, humeral width 4.0–4.6 mm. Body dark brown; head and pronotum covered with yellowish, tawny and brown pubescence, ventral surface with yellowish pubescence forming small spots sparsely scattered throughout. Head with four short tawny vittae behind upper eye lobes. Antennal scape, pedicel and 3^rd^ antennomere with sparse light yellowish pubescence, 4^th^ to 8^th^ antennomeres with same color pubescence at base, remainder with fine brown pubescence. Pronotum with two longitudinal tawny bands on each side of disk and other two longitudinal bands on lateral margins; the discal bands longer than two-thirds of pronotal length, sometimes interrupted anteromedially. Scutellum densely clothed with tawny pubescence, slightly sparse along middle. Elytron with pubescence predominantly brown, with tawny pubescence narrowly forming the subbasal annular marking, and some small spots scattered mainly near suture, with yellowish (or tawny) pubescence forming the middle band and the preapical stripe; the middle band usually moderately oblique, shape variable, widely interrupted to nearly interrupted near lateral margin, broadly and transversely reaching suture (in some individuals, the middle band complete, obliquely reaching suture directly without broadening and curving); the preapical stripe reduced, slightly shorter than one-fifth of elytral length. Legs (Fig. 31) clothed with sparse brown and dense yellowish pubescence of which the lighter one forming small spots moderately scattered on femora and becoming denser on tibiae.

Body elongate, oblong oval. Head (Fig. 22) with frons sparsely punctured; lower eye lobe 1.3 times as long as width, 0.8 times as long as gena. Antennae ca. 1.8–1.9 times as long as body length, surpassing elytral apex by 5–6 antennomeres; 3^rd^ antennomere ca. 1.8 times as long as scape, ca. 1.3 times as long as 4^th^ antennomere; scape and basal half of 3^rd^ antennomere coarsely punctured; scape to 3^rd^ antennomere sparsely fringed beneath. Pronotum 1.2 times as long as basal width, lateral spine developed, moderately thickened at base with acute apex; metasternum subequal in length to mesosternum. Elytra ca. 1.4 times as wide as pronotal base, 2.0 times as long as humeral width; humeri slightly constricted, widened at basal two-fifth, then convergent toward obliquely truncated apices; disk moderately punctured, becoming shallower at apical one-third, distinctly with moderate to large size, raised granules moderately sparse; subbasal tubercle developed, ca. 1.3 times as wide as scutellar width. Hindwings (Fig. 42) strongly reduced, 0.8 times as long as elytral length. Legs long and slender, metafemora slightly exceeding elytral apices.

**Male genitalia** (Figs 50, 60). Tergite VIII (Fig. 50a) transverse, slightly emarginated apically and rounded at sides, length 0.8 times as long as width. Tegmen (Fig. 50b–c) with lateral lobe widest at base, gently narrowed toward rounded apex. Median lobe (Fig. 50d–e) with apex subacute in antero-dorsal view. Endophallus (n = 3, Fig. 60) longer than triple length of median lobe, the length of MT ca. 2.5 times as long as the length of BPH, the length of CT+PB slightly longer than the length of BPH; MPH strongly curved at apical one-third, PB cylindrical at basal one-third, basal swelling (bs) of CT developed; APH moderately constrictive, ca. 0.6 times as wide as the maximum width of PB at base, with apical bulb (ab) heavily sclerotized apically, obliquely truncated in lateral view; small spicules densely distributed on apical bulb and anterior margin of PB.

**Female** (Figs 2, 3). Body length 17.2–20.7 mm, humeral width 5.0–5.6 mm. Almost identical to male in general appearance. Antennae ca. 1.3–1.4 times as long as body length, apical 3–4 antennomeres surpassing elytral apex; scape to 7^th^ antennomere fringed beneath; lower eye lobe subequal in length and width, 0.5 times as long as gena; pronotum subequal in length and basal width; elytron longer in proportion to body length (ca. 2.2 times as long as humeral width); legs comparatively short, metatibiae exceeding elytral apices at base.

#### Diagnosis.

Lower eye lobe rather short, 0.5 times as long as gena (in female); elytron with granules large and raised, sparsely scattered throughout, middle band variable, complete or interrupted to nearly interrupted near lateral margin; humeri slightly constricted; hindwings strongly reduced. Endophallus with APH constrictive, apical bulb (ab) heavily sclerotized apically, obliquely truncated in lateral view.

#### Distribution

(Map 1). China: Sichuan(?), Yunnan.

#### Remarks.

Based on the morphological similarities, the population from Gongshan County and Fugong County of Yunnan Province are considered as *Pseudoechthistatus granulatus* temporarily. The type locality of *Pseudoechthistatus granulatus*, “*Tatsienlu*”(= Kangding County) perceived to be doubtful for the following reasons. Based on reliable collecting data, the distribution of a flightless species (at least among this genus) does not support such remote distance (more than 400 km away from “*Tatsienlu*” to the population in Yunnan). Moreover, no individual has thus far been found in the intervening area. The population from Gongshan and Fugong could not be separated from the type specimen from “*Tatsienlu*” morphologically. The weak differences, such as slightly longer antennae and lighter pubescence color should be treated as intraspecific variation. Furthermore, females from Yunnan share the shorter lower eye lobes with the female type specimen, while other congeners have longer lower eye lobes (except for *Pseudoechthistatus pufujinae* sp. n.). The poor and handwritten label of the holotype (Fig. 19) is presumably simply mislabeled. In other words, “Tatsienlu” had been written on labels merely to indicate the general region, and the exact locality could be farther afield (Cox, 1945: 209, 212). Until now, no additional specimens have been reported or found from Kangding. (The first author had visited Kangding three times, trying hard to find topotype specimens but without success.) 5) Fugong was included in the distribution list by Li (1988, 2009) and Pu (1992). In order to clarify this doubt, further studies are necessary based on obtaining the topotype, especially the male specimens from Kangding.

### Pseudoechthistatus sinicus sp. n.

Taxon classificationAnimaliaColeopteraCerambycidae

http://zoobank.org/B402F732-1A65-41AE-B152-6C465BAB955E

Figures 9 , 10 , 24 , 34 , 43 , 52 , 59 , 71 , Map 1

#### Type material.

Holotype: male, “Yunnan, Dayao County, Santaixiang / Xiaobaicaoling / 2980 m 2013.V.29–30 / leg. Wen-Xuan Bi” (IZAS, IOZ(E) 1905347). Paratypes (22 males, 22 females): 3 males, 4 females, same data as holotype but (CBWX); 1 male, 1 female, same data as holotype but (SHEM); 1 male, 1 female, same data except “leg. Xiao-Dong Yang” (CCCC); 1 male, “CHINA. Yunnan, Binchuan / Jizushan / 2300 m 2010.VII.12 / leg. Xiao-Bin Song” (CBWX); 1 male, ditto except “2010.VII.16” (CSXB); 3 females, “ CHINA. Yunnan, Binchuan / Jizushan / 2258 m 2010.VI.10 / leg. Xiao-Dong Yang” (CCCC); 2 females, “CHINA, Yunnan, Dali zhou, / Binchuan county, Jizushan, / 2500–3200 m, 26.–31.VII.1993, / leg. C. Holzschuh” (CCH); 1 female, “Djo-Kou-La / alt. 1200 m / Nord Ouest Yunnan” (NHRS-JLKB000024084); 1 male, “YUNNAN 1800–2500 m / 25.10N 100.21E / WEISHAN mt. / 22–25.VI.92 / David Král leg.” (CCH); 10 males, 7 females, “CHINA. Yunnan, Weishan / Weibaoshan 2400–2500 m / 2015.VIII.16 / leg. Wen-Xuan Bi” (CBWX); 3 males, 3 females, ditto except “leg. Xiao-Dong Yang” (CCCC); 1 male, “Yunnan Yongping to Yangbi / 1955.V.29. / leg. Yang Xing-Chi”, “*Pseudoechthista*- / *tus obliquefasciatus* / *Pic* / det. Chiang Shu-Nan 19*61*”, “100” (IZAS, IOZ(E) 1905348).

#### Description.

**Figures 9–16.**
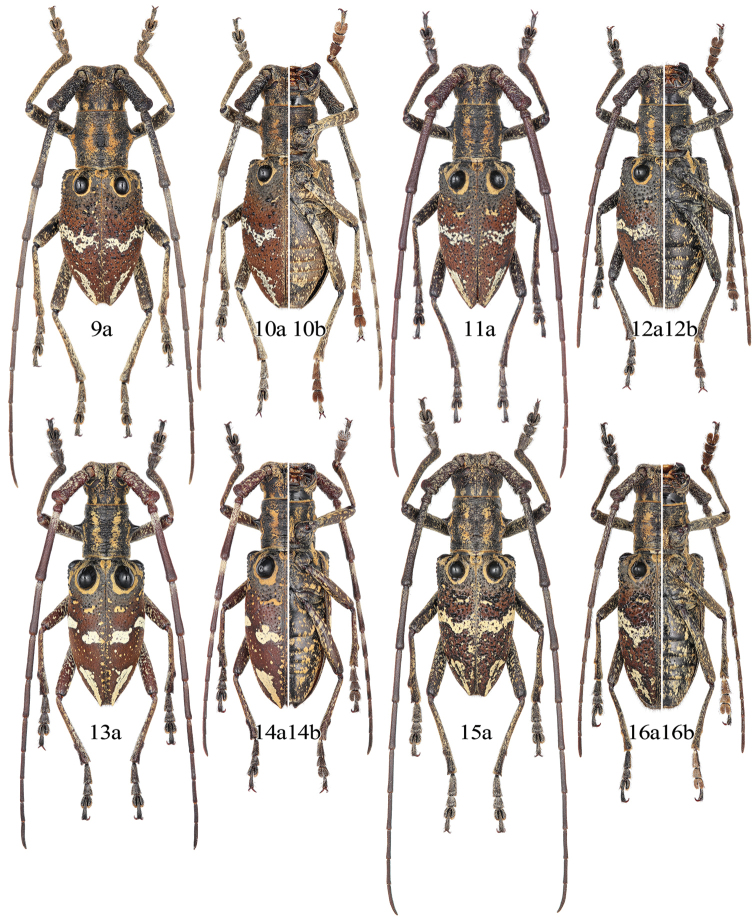
Habitus of *Pseudoechthistatus* spp. **9–10** *Pseudoechthistatus sinicus* sp. n. (paratype) **11–12** *Pseudoechthistatus chiangshunani* sp. n. (11 holotype, 12 paratype) **13-14** *Pseudoechthistatus holzschuhi* sp. n. (paratype) **15-16** *Pseudoechthistatus pufujiae* sp. n. (paratype) **9, 11, 13, 15** male **10, 12, 14, 16** female. **a** dorsal view **b** ventral view. Not to scale.

**Male** (Fig. 9). Body length 16.5–23.0 mm, humeral width 5.0–7.0 mm. Body dark brown; head, pronotum and ventral surface covered with tawny and brown pubescence. Head with four short tawny vittae behind upper eye lobes. Antennal scape with sparse light yellowish and brown pubescence; pedicel, basal two-thirds of 3^rd^ antennomere and basal half of 4^th^ antennomere with light yellowish pubescence, remainder with fine brown pubescence. Pronotum with two longitudinal tawny bands on each side of disk and other two longitudinal postmedian bands on lateral margins, the discal bands slightly longer than half of pronotal length. Scutellum densely clothed with tawny pubescence. Elytron with pubescence predominantly reddish brown, with tawny pubescence narrowly forming the subbasal annular marking and some small spots scattered near suture and humerus, with light yellowish pubescence forming the middle band and the preapical stripe; the middle band narrow, moderately oblique, irregularly marginated, zigzagged near middle, hardly reaching suture; the preapical stripe narrow, well developed. Legs (Fig. 34) densely clothed with tawny and brown pubescence of which the tawny one forming small spots sparsely scattered on femora and becoming denser on tibiae.

**Figures 17–20.**
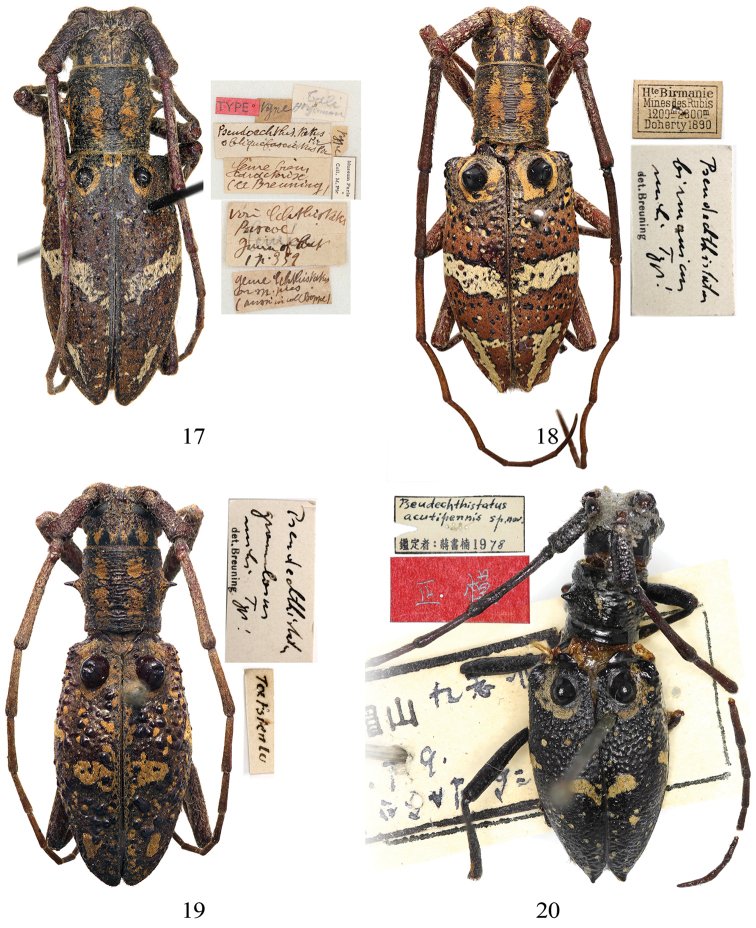
Habitus and label of the holotype of *Pseudoechthistatus* spp. **17** *Pseudoechthistatus obliquefasciatus* Pic, 1917 (female) **18** *Pseudoechthistatus birmanicus* Breuning, 1942 (male) **19** *Pseudoechthistatus granulatus* Breuning, 1942 (female) **20** *Pseudoechthistatus acutipennis* Chiang, 1981 (female).

**Figures 21–37.**
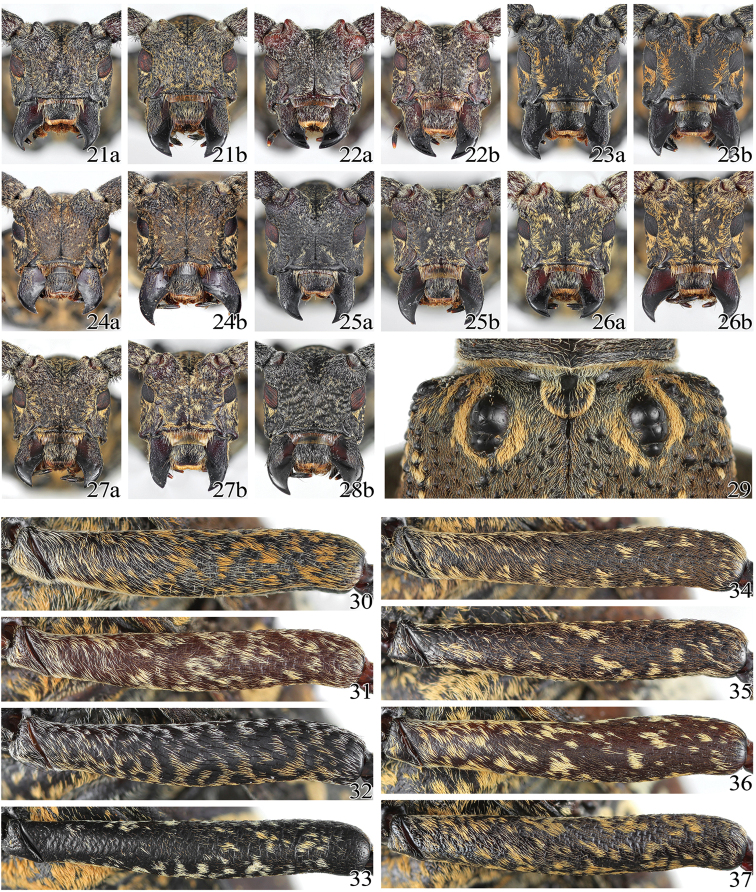
Habitus of *Pseudoechthistatus* spp. **21–28** head in frontal view **29** subbasal tubercle of elytron showing an abnormal form **30–37** femora in ventral view showing pubescence patterns **21, 29, 30** *Pseudoechthistatus obliquefasciatus* Pic, 1917 **22, 31** *Pseudoechthistatus granulatus* Breuning, 1942 **23, 33** *Pseudoechthistatus glabripennis* sp. n. **24, 34** *Pseudoechthistatus sinicus* sp. n. **25, 35** *Pseudoechthistatus chiangshunani* sp. n. **26, 36** *Pseudoechthistatus holzschuhi* sp. n. **27, 37** *Pseudoechthistatus pufujiae* sp. n. **28, 32** *Pseudoechthistatus acutipennis* Chiang, 1981. **a** male **b** female.

Body elongate, oblong oval. Head (Fig. 24) with frons sparsely and moderately punctured; lower eye lobe 1.1 times as long as width, 0.5 times as long as gena. Antennae ca. 1.6–1.7 times as long as body length, surpassing elytral apex at base of 6^th^ antennomere; 3^rd^ antennomere ca. 1.6 times as long as scape, ca. 1.3 times as long as 4^th^ antennomere; coarsely punctured on scape to basal half of 3^rd^ antennomere; scape to 3^rd^ antennomere fringed beneath. Pronotum subequal in length and basal width, lateral spine moderately long, thickened at base with acute apex; metasternum ca. 1.3 times as long as mesosternal length. Elytra 1.4 times as wide as pronotal base at humeri, 1.8 times as long as humeral width; humeri slightly constricted, widened at basal two-fifth, then convergent toward obliquely truncated apices; disk finely punctured, moderately granulated near humerus and behind basal one-fourth, weakened near apical one-third; subbasal tubercle moderately developed and raised, ca. 1.2 times as wide as scutellar width. Hindwings (Fig. 43) reduced, slightly shorter than elytral length.Legs long and slender, metafemora slightly exceeding elytral apices.

**Figures 38–48.**
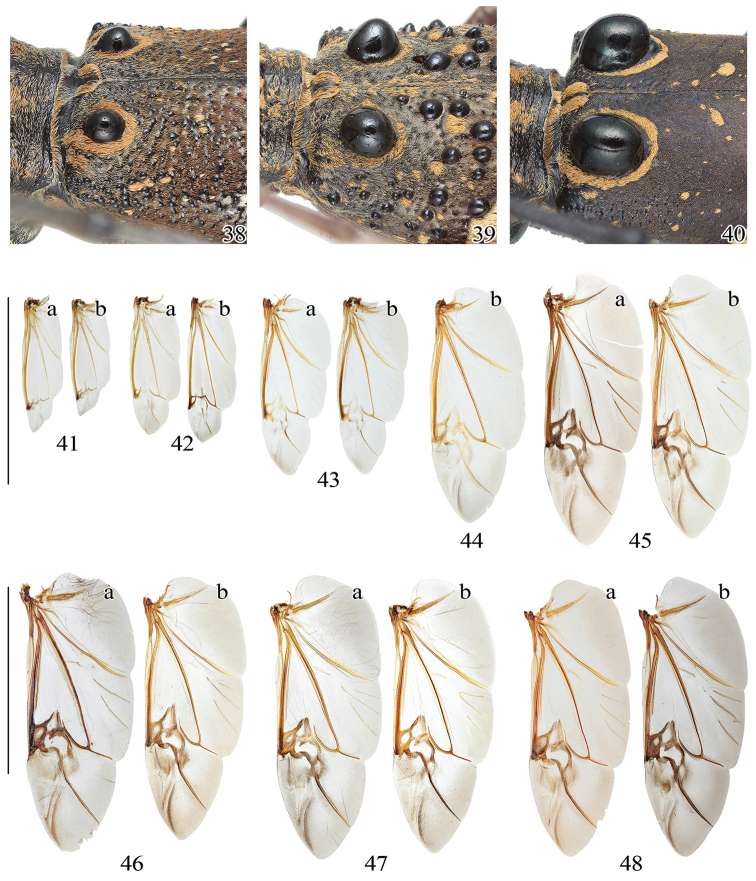
Habitus of *Pseudoechthistatus* spp (**a** male **b** female). **38–40** basal elytra in dorsal-lateral view showing shape and size of subbasal tubercles **41–48** hindwings of *Pseudoechthistatus* spp., scale = corresponding elytral length. **38, 41** *Pseudoechthistatus obliquefasciatus* Pic, 1917 **39, 42** *Pseudoechthistatus granulatus* Breuning, 1942 **43** *Pseudoechthistatus sinicus* sp. n. **44** *Pseudoechthistatus acutipennis* Chiang, 1981 **45** *Pseudoechthistatus pufujiae* sp. n. **46** *Pseudoechthistatus chiangshunani* sp. n. **47** *Pseudoechthistatus holzschuhi* sp. n. **40**, **48** *Pseudoechthistatus glabripennis* sp. n.

**Male genitalia** (Figs 52, 59). Tergite VIII (Fig. 52a) transverse, truncated apically and rounded at sides, length 0.8 times as long as width. Tegmen (Fig. 52b–c) with lateral lobe subparallel-sided in basal half, moderately narrowed toward acute apex. Median lobe (Fig. 52d–e) with apex acuminate in antero-dorsal view. Endophallus (n = 4, Fig. 59) subequal to triple length of median lobe, the length of MT ca. 2.4 times as long as the length of BPH, the length of CT+PB slightly longer than the length of BPH; MPH moderately curved at apical one-third, PB cylindrical at basal one-third, basal swelling (bs) of CT slightly swollen laterally; APH strongly constricted, ca. 0.5 times as wide as the maximum width of PB at base, with apical bulb (ab) heavily sclerotized apically, obliquely truncated in lateral view; small spicules densely distributed on apical bulb and apical two-fifths of PB.

**Figures 49–56.**
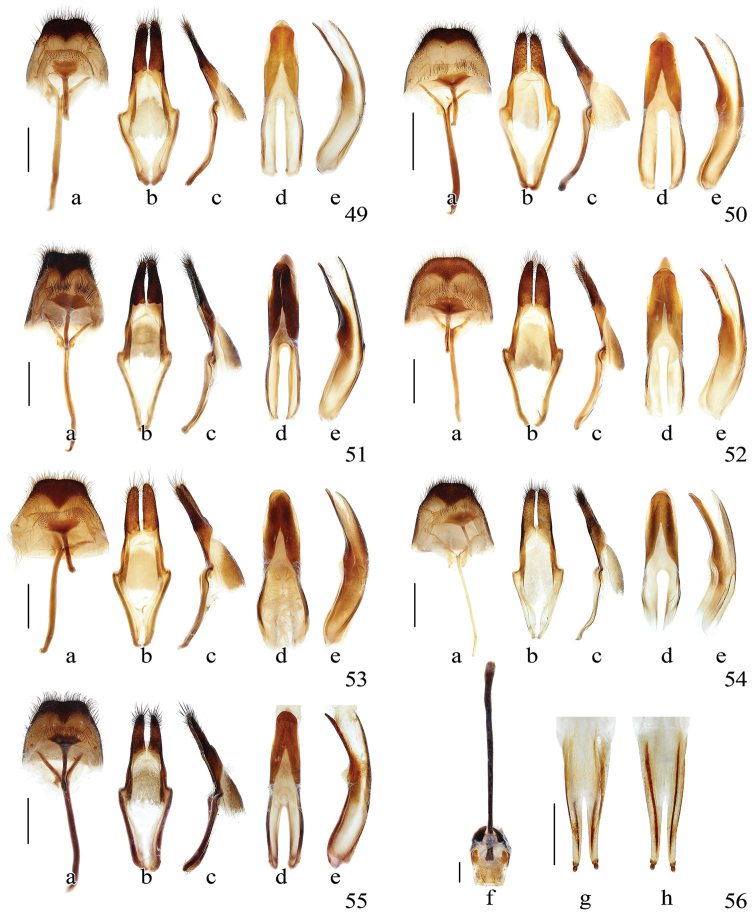
Terminalia of *Pseudoechthistatus* spp. **49, 56** *Pseudoechthistatus obliquefasciatus* Pic, 1917 **50** *Pseudoechthistatus granulatus* Breuning, 1942 **51** *Pseudoechthistatus glabripennis* sp. n. **52** *Pseudoechthistatus sinicus* sp. n. **53** *Pseudoechthistatus chiangshunani* sp. n. **54** *Pseudoechthistatus holzschuhi* sp. n. **55** *Pseudoechthistatus pufujiae* sp. n. **49–55** male. **a** tergite VIII with sternites VIII & IX **b** tegmen in ventral view **c** ditto in lateral view **d** median lobe in ventral view **e** ditto in lateral view **56** female. **f** sternite VIII **g** ovipositor in dorsal view **h** ditto in ventral view. Scale 1 mm.

**Figures 57–68.**
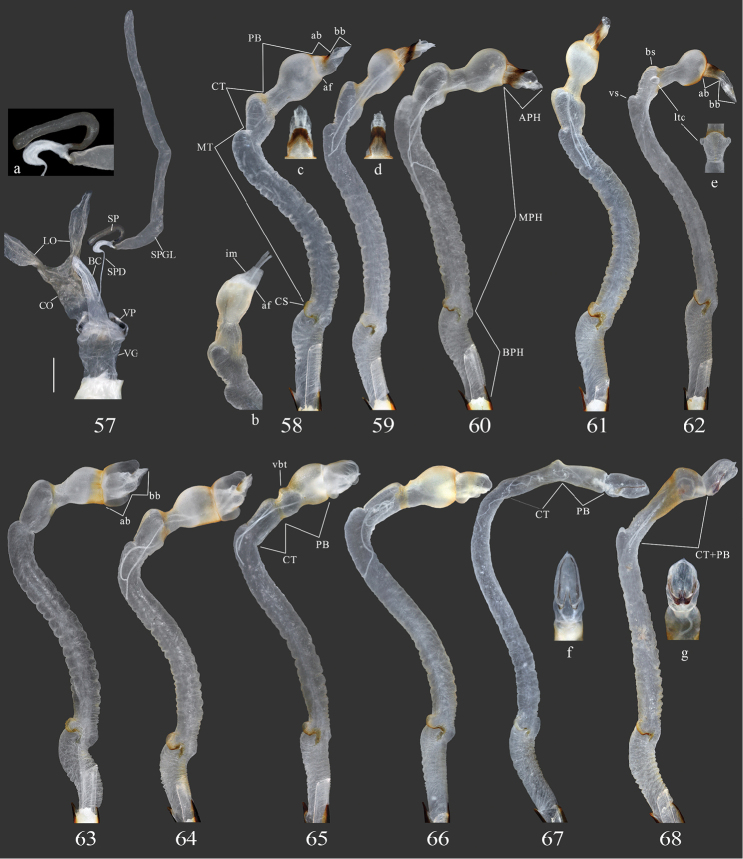
Terminalia of *Pseudoechthistatus* and *Paraleprodera* species. **57** spermatheca **58–67** endophallus in inflated and everted condition, lateral view **57–58** *Pseudoechthistatus obliquefasciatus* Pic **59** *Pseudoechthistatus sinicus* sp. n. **60** *Pseudoechthistatus granulatus* Breuning, 1942 **61** *Pseudoechthistatus pufujiae* sp. n. **62** *Pseudoechthistatus glabripennis* sp. n. **63** *Pseudoechthistatus holzschuhi* sp. n. **64** *Pseudoechthistatus chiangshunani* sp. n. **65** *Paraleprodera mesophthalma* Bi & Lin, 2012 **66** *Paraleprodera carolina* (Fairmaire, 1899) **67** *Paraleprodera triangularis* (Thomson, 1865) **68** *Paraleprodera diophthalma diophthalma* (Pascoe, 1857). **a** enlargement of spermathecal capsule (SP) **b** endophallus in inflated and non-everted condition, show internal membrane (im) of apical furrow (af) **c**, **d** APH in ventral view **e** CT in ventral view **f**, **g** APH in dorsal view.

**Figures 69–77.**
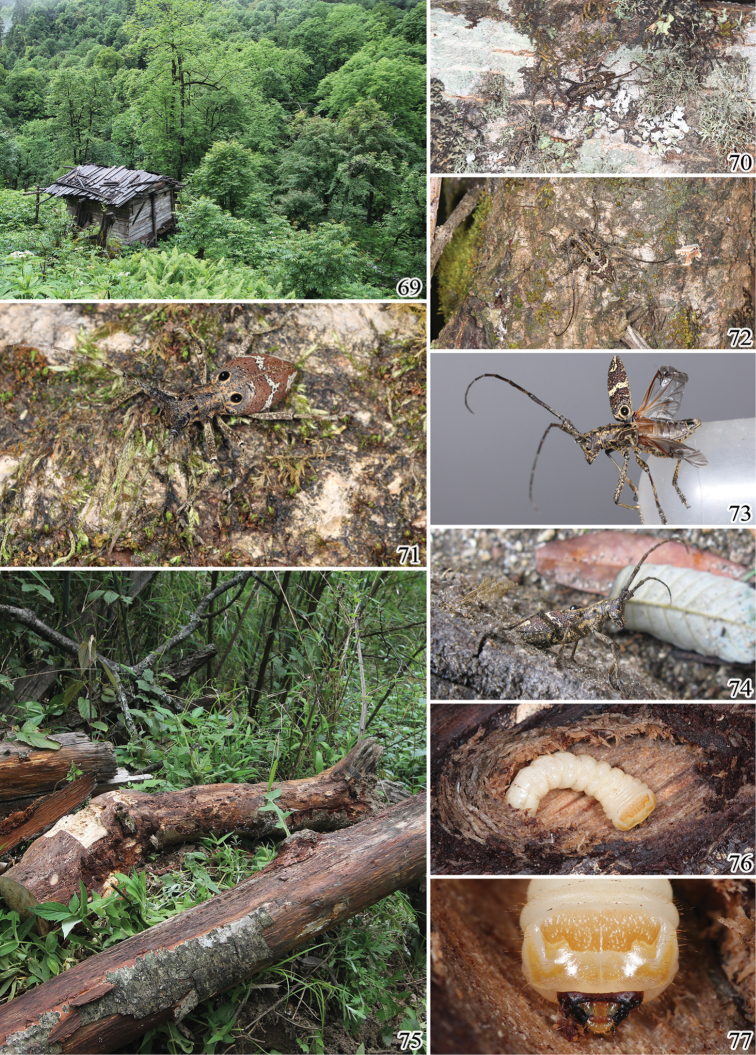
Biotope of *Pseudoechthistatus* species. **69–70** *Pseudoechthistatus granulatus* Breuning, 1942 **71** *Pseudoechthistatus sinicus* sp. n. **72–77** *Pseudoechthistatus pufujiae* sp. n.

**Female** (Fig. 10). Body length 17.0–22.0 mm, humeral width 5.4–6.6 mm. Almost identical to male in general appearance. Antennae ca. 1.2–1.3 times as long as body length, apical three or four antennomeres surpassing elytral apex; scape to 5^th^ or 6^th^ antennomere fringed beneath; 3^rd^ to 7^th^ antennomeres with light pubescence basally; elytron longer in proportion to body length (ca. 2.0 times as long as humeral width); legs comparatively short, metatibiae exceeding elytral apices at basal one-fourth.

#### Remarks.

This new species can be distinguished from most of the congeners by elytra comparatively shorter (only 1.8 times as long as humeral width), humeri distinctly constricted in both sexes; hindwings reduced; granules moderate in size near humerus and behind basal one-fourth, weakened near apical one-third; APH of endophallus strongly constricted. It is similar to *Pseudoechthistatus obliquefasciatus* by color pattern but can be easily distinguished by elytra relatively shorter in length, with bigger subbasal tubercles, median pubescent band not so oblique and APH of endophallus more strongly sclerited. It is similar to *Pseudoechthistatus granulatus* by size of elytral subbasal tubercles but can be separated by shorter elytra, weaker granules, and denser ventral tawny pubescence.

#### Etymology.

The new species is named after the country of its type locality.

#### Distribution

(Map 1). China: Yunnan.

### Pseudoechthistatus chiangshunani sp. n.

Taxon classificationAnimaliaColeopteraCerambycidae

http://zoobank.org/9FC642FC-C803-4C5B-B720-416FBC239D19

Figures 11 , 12 , 25 , 35 , 46 , 53 , 64 , Map 1

#### Type material.

Holotype: male,“Yunnan Prov., Zhengyuanxian / Jiujiazhen, Qianjiazhai / 2012.V.6 2600 m”, “N24°17.143’ / E101°15.060’ / leg. Ling-Zeng Meng ”(IZAS, IOZ(E) 1905352). Paratypes (16 males, 9 female): 1 male, “*Yunnan Jingdong*”, “*Paraleprodera opsiptera* *n. sp.* / det. Chen Shi-Xiang 19” (IZAS, IOZ(E) 1905346); 1 male, “*Yunnan Jingdong*”, “*Jingdong Wuliangshan* / *1800*–*2430 m.* / *1958.III.*” (IZAS, IOZ(E) 1905217); 2 males, “2009-VIII-1–3 / Yunnan, Zhenyuanxian, Jiujiaxiang / leg. Ji-Shan Xu & Jian-Xiong Zhang”, “N24.279°/ E101.264°/ Alt. 2160 m” (MHBU); 1 female, “CHINA. Yunnan, Yongde / Yalianxiang, Damaidi / 2175m 2012.V.8 / leg. Xiao-Dong Yang” (CCCC); 4 males, 1 female, “CHINA. Yunnan, Lincang / Manwanzhen, Shuibatoucun / Dahebadi 2103 m 2015.X.15 / leg. Bin Liu” (CLB); 1 female, ditto except “2113 m 2015.X.21” (CLB); 5 males, 3 females, ditto except “1950 m 2016.II.20–29 / local collector” (CLB); 1 male, 1 female, ditto except “1960 m 2016.I.23 / leg. Zi-Chun Xiong” (CZDY); 1 male, 1 female, ditto except “2016.II.10”(CZDY); 1 male, 1 female, ditto except “2016.III.21”(CZDY).

#### Description.

**Male.** (Fig. 11). Body length 16.0–24.3 mm, humeral width 4.9–7.9 mm. Body brownish black; head and pronotum covered with yellowish, tawny and brown pubescence, ventral surface with tawny pubescence forming small spots sparsely scattered throughout. Head with four tawny vittae on basal half of occiput behind upper eye lobes. Antennal scape, pedicel and basal one-fifth of 3^rd^ antennomere sparsely annularted with light yellowish pubescence, remainders with fine brown pubescence. Pronotum with two longitudinal tawny bands on each side of disk and other two longitudinal bands on lateral margins, the discal bands slightly longer than half of pronotal length. Scutellum densely clothed with tawny pubescence, slightly sparse along middle. Elytron with pubescence predominantly reddish brown, with tawny pubescence forming the subbasal annular marking, a short discontinuous transverse band at basal one-third near suture and a few small spots scattered near humerus and along suture, with light yellowish pubescence forming the middle band and the preapical stripe; the middle band narrow, moderately oblique, nearly interrupted at middle, zigzaged near suture, hardly reaching suture; the preapical stripe narrow, well developed. Legs (Fig. 35) clothed with sparse light brown and dense light yellowish pubescence of which the lighter one forming small spots moderately scattered.

Body elongate, oblong oval. Head (Fig. 25) with frons sparsely and coarsely punctured; lower eye lobe 1.2 times as long as width, 0.6 times as long as gena. Antennae ca. 1.6–1.7 times as long as body length, surpassing elytral apex at base of 6^th^ antennomere; 3^rd^ antennomere ca. 1.8 times as long as scape, ca. 1.3 times as long as 4^th^ antennomere; coarsely punctured on scape to 3^rd^ antennomere; scape to 4^th^ antennomere fringed beneath. Pronotum slightly longer than width at base, lateral spine developed, thickened at base with acute apex; metasternum ca. 1.5 times as long as mesosternal length. Elytra ca. 1.6 times as wide as pronotal base, 1.8 times as long as humeral width; subparallel-sided in basal two-thirds, slightly widened at basal half, then moderately convergent toward subacute apices; disk moderately and deeply punctured, moderately granulated near humerus to behind basal one-third, becoming indistinct subapically; subbasal tubercle strongly developed and raised, ca. 1.4 times as wide as scutellar width. Hindwings (Fig. 46) developed, ca. 1.4 times as long as elytral length. Legs long and slender, metafemora almost exceeding elytral apices.

**Male genitalia** (Figs 53, 64). Tergite VIII (Fig. 53a) transverse, slightly emarginated apically and straight sided, length 0.8 times as long as width. Tegmen (Fig. 53b–c) with lateral lobe subparallel-sided toward rounded apex. Median lobe (Fig. 53d–e) with apex rounded in antero-dorsal view. Endophallus (n = 4, Fig. 64) slightly longer than triple length of median lobe, the length of MT ca. 3.0 times as long as the length of BPH, the length of CT+PB slightly longer than the length of BPH; MPH strongly curved at apical two-fifths, PB cylindrical at basal one-fourth, basal swelling (bs) of CT moderately swollen anterolaterally; APH moderately swollen, slightly wider than the maximum width of PB at base, obliquely truncated in lateral view, with apical bubble (bb) provided with a pair of short rod-like sclerite subapically; small spicules evenly distributed on basal half of apical bulb and apical half of PB.

**Female** (Fig. 12). Body length 20.7–22.1 mm, humeral width 6.2–7.0 mm. Almost identical to male in general appearance. Antennae 1.2 times as long as body length, apical three antennomeres surpassing elytral apex; scape to 7^th^ antennomere fringed beneath; humeri slightly constricted; elytron longer in proportion to body length; legs comparatively short, metatibiae exceeding elytral apices at basal half.

#### Remarks.

This new species is similar to *Pseudoechthistatus sinicus* sp. n., but is distinguishable by having the hindwings fully developed, punctures on elytra distinct and deeper, and APH of endophallus swollen. It resembles *Pseudoechthistatus holzschuhi* but differs in having antennae (at least 3^rd^ to 4^th^ antennomeres) without yellowish annulations, 3^rd^ antennomere fringed beneath (fringed only at basal half in *Pseudoechthistatus holzschuhi*), punctures and granules on elytra more developed, elytra wider (compare with its length), humeri constricted in female. It can be distinguished from *Pseudoechthistatus birmanicus* by the longer 3^rd^ antennomere, deeper elytral punctures, smaller elytral granules and narrower median band on elytra, and the subbasal tubercle of elytron not so close to elytral base.

#### Etymology.

The new species is dedicated to the late Shu-Nan Chiang (1914–2013), an entomologist specialized in the taxonomy of Chinese Cerambycidae.

#### Distribution

(Map 1). China: Yunnan.

### Pseudoechthistatus holzschuhi sp. n.

Taxon classificationAnimaliaColeopteraCerambycidae

http://zoobank.org/C63ABD26-AF63-4390-8DA0-ACD8A41FE469

Figures 13 , 14 , 26 , 36 , 47 , 54 , 63 , Map 1

#### Type material.

Holotype: male, “CHINA. Yunnan, Jinping / Fenshuiling / 2311 m 2010.IX.18 / leg. Xiao-Dong Yang” (IZAS, IOZ(E) 1905353). Paratypes (8 males, 11 females): **China**: 1 female, same data as holotype except “2011.V.22” (CCCC); 1 female, ditto except 2011.V.26” (CCCC); 1 male, ditto except “2011.V.22 / leg. Jia-Hong Lin” (CCCC); 1 male, 1 female, “Yunnan Jinping Fenshuiling / 2010-VI-01 / leg. Wen-Hsin Lin 2250 m” (CJM); 1 female, “*Jinping* / *leg. Zeng Qing-Yao* / *1957.V*”, “Yunnan: *Jinping* / 195*7.V*”No. 56, host plant: fallen wood of *Quercus* sp. (IZAS, IOZ(E) 1905349); 1 female, “CHINA. Yunnan, Pingbian / Daweishan / 2000 m 2012.IX.28 / leg. Xiao-Dong Yang” (CCCC); **Vietnam**: 1 male, “VIETNAM. Lào Cai prov. / Sapa Mt. / 1600 m 2015.VII / local collector” (CBWX); 2 males 1 female, “May 2015; Vietnam / SAPA Mt. / 1800 m / native col. / Lao Cai” (CTT); 2 males, ditto except “June 2014” (CTT); 1 male, ditto except “June 2015” (CTT); 4 females, ditto except “July 2015” (CTT); 1 female, ditto except “September 2015” (CTT).

#### Description.

**Male** (Fig. 13). Body length 17.5–25.4 mm, humeral width 5.4–8.0 mm. Body dark brown; head, pronotum covered with yellowish, tawny and brown pubescence, ventral surface with light brown pubescence forming small spots sparsely scattered throughout. Head with four tawny vittae behind upper eye lobes of which the middle two are narrow and indistinct. Antennal scape and pedicel with sparse light yellowish pubescence; 3^rd^ and 4^th^ antennomeres annulate with light yellowish pubescence at basal one-fourth and becoming indistinct on 5^th^ to 7^th^ antennomeres, remainder with fine brown pubescence. Pronotum with two longitudinal tawny bands on each side of disk and another two longitudinal bands on lateral margins, the discal bands longer than two-thirds of pronotal length. Scutellum densely clothed with tawny pubescence. Elytron with pubescence predominantly brick-red, with tawny pubescence forming the subbasal annular marking, a short discontinuous transverse band at basal one-third near suture and some small spots sparsely scattered, becoming denser along suture, with light yellowish pubescence forming the middle band and the preapical stripe; the middle band moderately broad and oblique, interrupted or nearly interrupted at middle, transversely reaching suture; the preapical stripe well developed, moderately broader at base. Legs (Fig. 36) clothed with sparse brown and dense light yellowish pubescence of which the lighter one forming small spots moderately scattered on femora and becoming denser on tibiae.

Body elongate, oblong oval. Head (Fig. 26) with frons sparsely and finely punctured; lower eye lobe subequal in length and width, 0.6 times as long as gena. Antennae ca. 1.5–1.7 times as long as body length, surpassing elytral apex at base of 6^th^ antennomere; 3^rd^ antennomere ca. 1.7 times as long as scape, ca. 1.3 times as long as 4^th^ antennomere; scape coarsely punctured; scape to basal half of 3^rd^ antennomere fringed beneath. Pronotum 1.1 times as long as basal width, lateral spine developed, thickened at base with acute apex; metasternum ca. 1.3 times as long as mesosternal length. Elytra ca. 1.6 times as wide as pronotal base, 1.9 times as long as humeral width; subparallel-sided in basal half, then moderately convergent toward subacute apices; disk sparsely and finely punctured at basal half, becoming shallower posteriorly, sparsely granulated behind humerus, granules hardly reaching basal one-third; subbasal tubercle strongly developed and raised, ca. 1.7 times as wide as scutellar width. Hindwings (Fig. 47) developed, ca. 1.4–1.5 times as long as elytral length. Legs moderately long and slender, metatibiae exceeding elytral apices at basal one-fourth.

**Male genitalia** (Figs 54, 63). Tergite VIII (Fig. 54a) slightly wider than long, truncated apically and rounded at sides. Tegmen (Fig. 54c–d) with lateral lobe subparallel-sided toward rounded apex. Median lobe (Fig. 54e–f) with apex acuminate in antero-dorsal view. Endophallus (n = 3, Fig. 63) longer than triple length of median lobe, the length of MT ca. 2.3 times as long as the length of BPH, the length of CT+PB subequal to the length of BPH, CT slightly longer than PB; MPH moderately curved at apical two-fifth, PB cylindrical at basal one-fourth, basal swelling (bs) of CT moderately swollen anterolaterally; APH moderately swollen, slightly wider than the maximum width of PB at base, obliquely truncated in lateral view; small spicules evenly distributed on basal half of apical bulb, densely distributed on apical one-third of PB.

**Female** (Fig. 14). Body length 19.4–23.0 mm, humeral width 6.7–7.4 mm. Almost identical to male in general appearance. Antennae 1.2 times as long as body length, surpassing elytral apex at base of 9^th^ antennomere; basal 7 antennomeres fringed beneath; 3^rd^ to 6^th^ antennomeres distinctly annulate with light yellowish pubescence at base; elytron longer in proportion to body length; legs comparatively short, metatibiae exceeding elytral apices at apical two-third.

#### Remarks.

This new species is most similar to *Pseudoechthistatus birmanicus* and *Pseudoechthistatus chiangshunani* sp. n. by the general habitus but can be distinguished from the former by the elytral granules being rather weakly developed and limited within basal one-third; elytral punctures finer and sparser; middle band of elytron interrupted or nearly interrupted and more developed pronotal lateral spines. It can also be distinguished from the latter by the antenna being shorter than body length, at least 3^rd^ to 4^th^ antennomeres with light yellowish pubescent annulations at base; elytra relatively smooth, granulate only at basal one-third, elytral punctures finer and sparser; female humeri similar to male, not constricted.

#### Etymology.

The new species is named after Carolus Holzschuh, a specialist in Cerambycidae, who kindly provided his collection for this study.

#### Distribution

(Map 1). China: Yunnan; Vietnam: Lào Cai.

### Pseudoechthistatus pufujiae sp. n.

Taxon classificationAnimaliaColeopteraCerambycidae

http://zoobank.org/CF721CCB-5265-4ED0-9A63-27A466090FEE

Figures 15 , 16 , 27 , 37 , 45 , 55 , 61 , 73–77 , Map 1

Pseudoechthistatus birmanicus : Pu 1992: 601. (*nec* Breuning, 1942).

#### Type material.

Holotype: male, “CHINA. Yunnan, Lushui / Yaojiaping 2450 m / 2015.V.4 em. VI.9 / leg. Wen-Xuan Bi”, “IOZ(E)1905345” (IZAS). Paratypes (5 males, 6 females): 2 males, 1 female, same data as holotype but (CBWX); 1 male, ditto except “em. VI.4” (CBWX); 1 male, ditto except “2015.VIII.13” (CBWX); 1 female, “Yunnan Lushui / Yaojiaping 2500 m”, “1981.VI.2 / leg. Wang Shu-Yong”, “*Pseudechthistatus* / *birmanicus* / *Breuning* / det. Pu Fu-Ji 19”, “IOZ(E)1905350” (IZAS); 1 female, “CHINA. Yunnan, Lushui / Yaojiaping / 2700 m 2010.VI.21 / leg. Wen-Xuan Bi” (CBWX); 1 female, ditto except “2600m 2010.VI.23” (CBWX); 1 female, ditto except “2700 m 2010.VI.21 / leg. Xiao-Dong Yang” (CCCC); 1 male, “Yunnan, Lushui, Pianma / Gangfang alt.2402 m / 2014.IV.11 night / leg. Xuan-Kong Jiang, Tian Lu”, “25°17.776'N / 98°45.862'E / YNGLGS-14-36”, “IOZ(E)1905345” (IZAS); 1 female, “CHINA. Yunnan, Baoshan / Baihualing 2350m / 2015.V.4 em. VII.1 / leg. Wen-Xuan Bi” (CBWX).

#### Description.

Male (Fig. 15). Body length 19.0–23.5 mm, humeral width 6.0–7.5 mm. Body dark brown; head, pronotum covered with tawny and brown pubescence, ventral surface with yellowish to light brown pubescence of which the lighter one forming small spots sparsely scattered throughout. Head with four tawny vittae behind upper eye lobes distinctly. Antennal scape, pedicel, basal two-thirds of 3^rd^ antennomere and basal half of 4^th^ antennomere with light yellowish pubescence, remainder with fine brown pubescence. Pronotum with two longitudinal tawny bands on each side of disk and other two indistinct longitudinal bands on lateral margins, the discal bands slightly longer than half of pronotal length. Scutellum densely clothed with tawny pubescence, slightly sparse along middle. Elytron with pubescence predominantly brick-red, with tawny pubescence forming the subbasal annular marking and some small spots scattered near suture and behind humerus, with light yellowish pubescence forming a short transverse band at basal one-fourth near suture, with the same pubescence forming the middle band and the preapical stripe; the middle band moderately broad and oblique, complete, slightly curved or strongly zigzagged near suture and reaching suture; the preapical stripe moderately broader at base. Legs (Fig. 37) densely clothed with intermixed tawny and light brown pubescence.

Body elongate, oblong oval. Head (Fig. 27) with frons sparsely and coarsely punctured; lower eye lobe subequal in length and width, 0.7 times as long as gena. Antennae ca. 1.7–1.8 times as long as body length, surpassing elytral apex at base of 6^th^ antennomere; 3^rd^ antennomere ca. 1.9 times as long as scape, ca. 1.3 times as long as 4^th^ antennomere; coarsely punctured on scape to basal half of 3^rd^ antennomere; scape to 3^rd^ or 4^th^ antennomere fringed beneath. Pronotum subequal in length and basal width, lateral spine very short, slightly thickened at base with acute apex; metasternum ca. 1.5 times as long as mesosternal length. Elytra ca. 1.5 times as wide as pronotal base, 1.8 times as long as humeral width; subparallel-sided in basal half, then moderately convergent toward subacute apices; disk deeply and coarsely punctured, sparsely and slightly granulated near humerus and scutellum; subbasal tubercle moderately developed and raised, ca. 1.2 times as wide as scutellar width. Hindwings (Fig. 45) developed, ca. 1.4 times as long as elytral length. Legs moderately long and slender, metafemora slightly exceeding elytral apices.

**Male genitalia** (Figs 55, 61). Tergite VIII (Fig. 55a) transverse, slightly emarginated apically and rounded at sides, length 0.9 times as long as width. Tegmen (Fig. 55b–c) with lateral lobe widest at base, gently narrowed toward subacute apex. Median lobe (Fig. 55d–e) with apex subacute in antero-dorsal view. Endophallus (n = 2, Fig. 61) subequal to triple length of median lobe, the length of MT ca. 2.5 times as long as the length of BPH, the length of CT+PB subequal to the length of BPH; MPH moderately curved at apical one-third, PB cylindrical at basal one-third, basal swelling (bs) of CT moderately swollen anterolaterally; APH strongly constricted, ca. 0.4 times as wide as the maximum width of PB at base, with apical bulb (ab) heavily sclerotized in apical half, obliquely truncated in lateral view; small spicules moderately distributed on apical bulb and apical one-third of PB.

**Female** (Fig. 16). Body length 18.2–22.7 mm, humeral width 5.5–7.0 mm. Almost identical to male in general appearance. Antennae ca. 1.2 times as long as body length, apical 3 antennomeres surpassing elytral apex; scape to 6^th^ or 7^th^ antennomere fringed beneath; lower eye lobe subequal in length and width, 0.4 times as long as gena; elytron longer in proportion to body length; legs comparatively short, metatibiae exceeding elytral apices at basal two-third.

#### Remarks.

This new species can be distinguished from most of the congeners (except *Pseudoechthistatus acutipennis*) by elytral disk deeply and coarsely punctured throughout and limited granulated near base. It can be easily distinguished from *Pseudoechthistatus acutipennis* by pronotal bands and elytral preapical stripe developed, elytral apices subacute, hindwings developed (in *Pseudoechthistatus acutipennis*, pronotal bands reduced, preapical stripe of elytron absent, elytral apices strongly acute and hindwings reduced).

Pu (1992) misidentified this species as *Pseudoechthistatus birmanicus*, since the original description of the latter was too simple. Based on the type pictures, *Pseudoechthistatus birmanicus* can be easily separated from this new species by elytra with bigger and flattened granules from base to near apex, while elytral punctures finer. The middle band of the elytron is variable in the new species and cannot be used for a reliable diagnosis.

#### Etymology.

The new species is dedicated to the late Fu-Ji Pu (1932–2002), a specialist in Chinese Cerambycidae.

#### Distribution

(Map 1). China: Yunnan.

### Pseudoechthistatus glabripennis sp. n.

Taxon classificationAnimaliaColeopteraCerambycidae

http://zoobank.org/8CF864B0-AC72-46B6-946E-E2BD976E23DB

Figures 7 , 8 , 23 , 33 , 40 , 48 , 51 , 62 , Map 1

#### Type material.

Holotype: male, “CHINA. Yunnan / Menglun, 55 km / 650 m 2012.IV.25 / leg. Chao Wu” (IZAS, IOZ(E) 1905354). Paratypes (8 males, 9 females): **China**: 1 female, “Yunnan, Pingbian, Daweishan / peak, 2013.VIII.15 / leg. Chun-Xiang Liu & Kai-Qin Li”, “2094 m light trap / 22°54'23.1"N, / 103°41'48.5"E (IZAS, IOZ(E) 1905351); 1 male, “CHINA. Yunnan, Pingbian / Daweishan / 2100 m 2010.V.20 / leg. Wen-Hsin Lin” (CCCC); 1 male, ditto except “2093 m 2012.IX.27 / leg. Xiao-Dong Yang” (CCCC); 1 female, ditto except “2090 m 2011.VI.11” (CCCC); 1 female, ditto except “2011-VI-11” (CJM); 1 male, 2 females, ditto except “2129 m 2016.IV.20” (CCCC); 1 female, ditto except “2013.V.13 / leg. Chao Li light trap” (CLC); 1 male, 2 females, “Yunnan Honghezhou Pingbian / Daweishan 2015.V.21 / leg. Tian-Long He”, “22.551172°N / 103.415424°E / 1989 m observe” (CHTL); 2 males, 1 female, “Yunnansheng, Honghezhou, Pingbianxian / Daweishan Ziranbaohuqu / 2015.V.18 / Tian-Long He leg.” (CGQH); **Vietnam**: 1 male, “VIETNAM: Cao Bang Prov. / Phia-Oac Mtn. road, 1800 m / 22°36.914'N, 105°51.798'E / 2 May 2012 - sweeping / S. W. Lingafelter”(NMNH); 1 male, ditto except “on road (day) / Eduard Jendek, coll.”(NMNH).

#### Description.

Male. (Fig. 7). Body length 22.0–25.6 mm, humeral width 6.7–7.4 mm. Body brownish black; head, pronotum covered with tawny and brown pubescence, ventral surface with tawny pubescence and forming two discontinuous longitudinal bands on each side of abdomen. Head with a pair of tawny vittae on each side of occiput and reaching apical margin of vertex. Antennal scape, pedicel and basal one-fourth of 3^rd^ antennomeres moderately covered with light yellowish pubescence, remainder covered with fine brown pubescence. Pronotum with two longitudinal tawny bands on each side of disk and other two longitudinal postmedian bands on lateral margins, the discal bands about four-fifths as long as pronotal length. Scutellum densely clothed with tawny pubescence, except a narrow median glabrous line. Elytron with dark purple sheen, with tawny pubescence narrowly forming the subbasal annular marking and some small spots scattered mainly near suture, with yellowish pubescence forming the middle band and the preapical stripe, remainder with very fine pubescence; the middle band moderately oblique, complete, regularly shaped, nearly reaching suture; the preapical stripe narrow, slightly longer than one-fourth of elytral length. Legs (Fig. 33) clothed with sparse brown and dense light yellowish pubescence of which the lighter one forming small spots sparsely scattered.

Body elongate, fusiform. Head (Fig. 23) with frons sparsely and finely punctured; lower eye lobe 1.3 times as long as width, 0.6 times as long as gena. Antennae ca. 1.7–1.8 times as long as body length, surpassing elytral apex by six antennomeres; 3^rd^ antennomere ca. 1.8 times as long as scape, ca. 1.3 times as long as 4^th^ antennomere; coarsely punctured on scape to 3^rd^ antennomere; scape to 3^rd^ antennomere fringed beneath. Pronotum 1.2 times as long as basal width, lateral spine short, slightly thickened at base with acute apex; metasternum ca. 1.5 times as long as mesosternal length. Elytra 1.6 times as wide as pronotal base, 2.1 times as long as humeral width; distinctly widest across humeri, then strongly convergent toward subacute apices; disk smooth, very finely punctured, moderately granulated near humerus; subbasal tubercle strongly developed and raised, ca. 1.4–1.6 times as wide as scutellar width. Hindwings (Fig. 48) developed, ca. 1.5 times as long as elytral length. Legs long and slender, metafemora almost exceeding elytral apices.

**Male genitalia** (Figs 51, 62). Tergite VIII (Fig. 51a) slightly longer than width, slightly emarginated apically and straight sided. Tegmen (Fig. 51b–c) with lateral lobe widest at base, gently narrowed toward apical half then straightly toward rounded apex. Median lobe (Fig. 51d–e) with apex roundly acuminate in antero-dorsal view. Endophallus (n = 4, Fig. 62) slightly longer than triple length of median lobe, the length of MT ca. 2.1 times as long as the length of BPH, the length of CT+PB slightly shorter than the length of BPH; MPH moderately curved at apical one-fourth, PB cylindrical at basal half, basal swelling (bs) of CT well developed; APH strongly constrictive, ca. one half as wide as the maximum width of PB at base, with apical bulb (ab) slightly sclerotized ventrally, subcylindrical in lateral view; small spicules densely distributed on apical bulb, apical margin and dorsal surface of PB.

**Female** (Fig. 8). Body length 24.0–25.1 mm, humeral width 7.3–7.4 mm. Almost identical to male in general appearance. Antennae ca. 1.2 times as long as body length, apical 3 antennomeres surpassing elytral apex; scape to 6^th^ antennomere fringed beneath; pronotum subequal in length and basal width; elytra subparallel-sided in basal half; elytron longer than males in proportion to body length; legs comparatively short, metatibia exceeding elytral apices at basal two-third.

#### Remarks.

This new species is easily distinguishable from congeners by combination of the following characters: elytral disk smooth, very finely punctured and pubescent, with dark purple sheen; middle band of elytron moderately oblique, complete; elytra distinctly widest across humeri (at least in males). Endophallus with the overall shape unique, especially by basal swelling (bs) of CT distinctly tuberculate laterally; APH strongly constrictive and subcylindrical in lateral view.

#### Etymology.

The new species is named from a combination of the Latin stem, ‘glabri’and ‘pennis’referring to the smooth surface of elytra.

#### Distribution

(Map 1). China: Yunnan; Vietnam: Cao Bằng.

##### Biological notes

No biological information has been so far available for *Pseudoechthistatus*. This overview is based on notes from several collectors and the observation of the first author as well as the label data from the specimens. Most species appear to occur in broadleaf deciduous or mixed coniferous and broadleaf forests (Fig. 69) at high elevations between 1800–3000 m, with the exception of an individual of *Pseudoechthistatus glabripennis* collected at 650 m, the lowest elevation known for this genus.

Adults were mostly observed on dead leaves and branches: *Pseudoechthistatus granulatus* were feeding on dead leaves or bark of *Pterocarya* sp. (Juglandaceae) and *Acer* spp. (Aceraceae); the population of *Pseudoechthistatus chiangshunani* from Manwanzhen, Lincang City were crawling on the trunk of dead *Juglans regia* (Juglandaceae) or feeding on dead leaves of *Alnus cremastogyne* (Betulaceae); some specimens of *Pseudoechthistatus sinicus* and *Pseudoechthistatus obliquefasciatus* were collected by beating dead branches of *Cyclobalanopsis* spp. and *Quercus* spp. (Fagaceae), while the population of *Pseudoechthistatus sinicus* in Xiaobaicaoling, Santaixiang, Dayao County, were feeding on living leaves of *Acer* sp. Some adults of *Pseudoechthistatus pufujiae* were reared from larvae collected under bark of a fallen tree of *Pterocarya* (Figs 75–77) in Yaojiaping, some larvae of *Pseudoechthistatus granulatus* were found in the same tree species in Gongshan but failed to emerge. One adult of *Pseudoechthistatus chiangshunani* was found in its pupal cell in a partly rotten wood of *Alnus cremastogyne* (Betulaceae). Two possible larvae of *Pseudoechthistatus granulatus* and *Pseudoechthistatus sinicus*, which were collected under bark of conifers but died due to the high temperature at lower elevation, were preserved properly for a further study.

Most species are nocturnal, and remain hidden in or around their host plants during daytime (Figs 71–72). Most specimens were collected by observing, beating, sweeping vegetation or by using light traps. Some individuals of *Pseudoechthistatus sinicus* were observed crawling on ground in the daytime. One female of *Pseudoechthistatus pufujiae* laying eggs on a fallen log was observed at noon (Fig. 74).

Besides the species with reduced hindwings which are apparently flightless, *Pseudoechthistatus pufujiae* (Fig. 73) with normal hindwings was observed flying only short distances when disturbed in the lab or in the field. A series of *Pseudoechthistatus glabripennis* attracted to a light trap indicates strong flying ability of that species.

Up to now, only *Pseudoechthistatus glabripennis* and *Pseudoechthistatus holzschuhi*, both with normally developed hindwings, are known to be sympatric in Fenshuiling, southeast Yunnan (Map 1). Those species with reduced hindwings appear to be allopatric. The Gongshan population of *Pseudoechthistatus granulatus* appears to be close to the northern population of *Pseudoechthistatus obliquefasciatus*, which is actually separated by the Nushan Mountains. *Pseudoechthistatus obliquefasciatus* is not known to be sympatric with *Pseudoechthistatus sinicus*, but the type locality of *Pseudoechthistatus obliquefasciatus* cannot be precisely localized (see the remarks of *Pseudoechthistatus obliquefasciatus*).

*Pseudoechthistatus sinicus* and hunting spiders (possibly Lycosidae) were active on ground vegetation simultaneously at night (observed in Weibaoshan and Xiaobaicaoling). In consideration of the elytral subbasal tubercles of *Pseudoechthistatus* that resemble the posterior median eyes of the spiders, we suppose this resemblance may represent a case of Batesian mimicry, but more evidence is required before any conclusion can be reached.

## Discussion

The shortened metasternum (associated with reduced hindwings) was one of the diagnostic characters used to define Dorcadionini, Morimopsini, Parmenini, and Phrissomini of Lamiinae (Breuning 1950), and was followed by most subsequent authors (e.g. Gressitt 1951, Rondon and Breuning 1970). However, such an arbitrarily selected character has likely evolved many times and has been noted in many clearly distantly related genera and is therefore unsatisfactory for tribal classification (Švácha and Lawrence 2014). Sama (2008) synonymized Phrissomini and Dorcadionini with Lamiini which is acceptable, while another, probably polyphyletic, tribe Morimopsini needs further study.

In Breuning’s tribal system of the Lamiinae, the occurrence of species with complete and reduced hindwings within the same genus or subgenus is uncommon, e.g. subgenus *Pseudale* of *Pterolophia* (Malihara 1988; Yamasako 2016 pers. comm.) and *Spalacopsis* (Tyson 1973; Lingafelter pers. comm.). In another instance, treating winged species under *Pseudoechthistatus* is supported not only by the similarities of external features (except the metasternum and its related characteristics) but also by the resemblances of the endophallic structures. The morphology of the endophallus is therefore considered useful for distinguishing and/or defining taxa of *Pseudoechthistatus* as well as other Lamiinae (e.g. Ehara 1954, Nakamine and Takeda 2008).

Investigation of the inflated endophallus in Cerambycidae was considered to have been undertaken for the first time recently (Danilevsky et al. 2005); however, Kuboki (1980, 1981) probably was the first person who investigated several lepturine species and pointed out the taxonomic significance of the structure of the endophallus. Although only an abbreviated word “everted” was presented in the paper, he in fact established a complicated way to evert and inflate the endophallus (Kuboki 2016 pers. comm.). His work has been ignored, as well as the voluminous non-English literature that has never been translated. In contrast, the endophallus in an uninflated condition has been more widely studied (e.g. Lingafelter and Hoebeke 2002).

The previous studies on the inflated endophallus can be subdivided into two paths (Yamasako and Ohbayashi 2012a): Danilevsky et al. (2005), Danilevsky and Kasatkin (2006), Kasatkin (2006), Ohbayashi and Bi (2014), Bi and Ohbayashi (2015), etc. investigated the endophallus in its everted condition; while Yamasako and Ohbayashi (2011, 2012b, 2012c), Yamasako (2014), Yamasako and Chou (2014a, 2014b), Bi and Lin (2014) etc. studied the endophallus in a non-everted condition. Yamasako and Ohbayashi (2012a) compared the advantages and the disadvantages of both conditions and concluded that observation of the endophallus is desirable in the “inflated and everted” condition, but the non-everted condition is useful for many taxa because it shows similar character states when there is no sclerotized structure supporting the membranous parts. In our study on *Pseudoechthistatus* and its relatives, however, the developed internal membrane, the sclerotized apical bulb or the presence of sclerite in apical phallomere make the APH hidden inside the endophallus in the non-everted condition and no critical structures of apical phallomere can be observed (Fig. 58b). Thus, a better comparison of endophallic structure is proposed to be done in the everted condition at least in Lamiini *sensu lato*. Of course, the technique to evert the endophallus still needs improvement, although a preliminary method is provided by Rubenyan (2002).

The endophallic terminology of Cerambycidae has been proposed and applied for various taxa by several authors (e.g. Danilevsky et al. 2005 for Dorcadionini; Kasatkin 2006 modified for family; Yamasako and Ohbayashi 2011 for Mesosini). Even so, the complex and individual structure of the endophallus among the family are still difficult to define congruously. The definition of PB for *Paraleprodera mesophthalma* (Lamiini-Monochamini) in Bi and Lin (2012) is revised in this paper for a consistent comparison to its congeners and to *Pseudoechthistatus*.

Finally, endophallic structures of twelve species or subspecies of *Paraleprodera* Breuning, 1935 have been investigated for comparison with *Pseudoechthistatus* Pic, 1917 in this study. As a result, *Pseudoechthistatus* can be clearly distinguished from *Paraleprodera* (see generic diagnosis). However, the endophallic diversity of *Paraleprodera* is considerable and the genus may be subdivided into at least two groups: the *triangularis* group containing *Paraleprodera bigemmata*, *Paraleprodera bisignata*, *Paraleprodera diophthalma* with subspecies and *Paraleprodera triangularis*, which are characterized by CT less developed (without a distinct swelling) and APH with a pair of U-shaped sclerites (Figs 67-f, 68-g) (*Paraleprodera crucifera*, the type species of *Paraleprodera* which is morphologically similar to *Paraleprodera triangularis*, probably also belongs to this group); the *carolina* group containing *Paraleprodera carolina*, Paraleprodera cf. flavoplagiata, *Paraleprodera itzingeri*, *Paraleprodera mesophthalma*, and *Paraleprodera stephanus*, which are characterized by CT swollen posteroventrally and PB bearing a ventral tubercle (vbt) (Fig. 65). The endophallus of *Paraleprodera insidiosa* resembles neither of the above groups. The endophallic structure of the *carolina* group has a much closer resemblance to *Pseudoechthistatus* than to the *triangularis* group, indicating that *Paraleprodera* might be para- or polyphyletic, which is also supported by its variable pronotal structures. However, these considerations are beyond the scope of this paper, and the generic treatment requires a thorough study in the future.

### Key to the species of *Pseudoechthistatus*

**Table:** 

1	Elytral disk smooth, very finely punctured; elytra distinctly widest at humeri (at least in males)	***Pseudoechthistatus glabripennis* sp. n.**
–	Elytral disk distinctly punctured and/or granulated; elytra widest near middle or subparallel in basal half	**2**
2	Pronotal longitudinal pubescent bands reduced, shorter than one-third of pronotal length; elytron strongly pointed apically, preapical stripe absent	***Pseudoechthistatus acutipennis***
–	Pronotal longitudinal pubescent bands well developed, longer than half of pronotal length; elytron rounded or obliquely truncated to subacute apically, with a more or less distinct preapical stripe	**3**
3	Elytral humeri distinctly narrower, elytra widened near middle; hindwings strongly reduced, distinctly shorter than elytral length	**4**
–	Elytra subparallel in basal half (at least in males); hindwings developed, distinctly longer than elytral length	**6**
4	Subbasal tubercle of elytron moderate in size, subequal to or slightly narrower than scutellar width; elytral middle band strongly oblique, more than 40 degrees to transverse axis	***Pseudoechthistatus obliquefasciatus***
–	Subbasal tubercle of elytron large, at least 1.2 times wider than scutellar width; elytral middle band moderately oblique or nearly transverse, less than 30 degrees to transverse axis	**5**
5	Elytra long, 2.0 times (in males) or 2.2 times (in females) as long as humeral width; elytral disk sparsely covered with large and raised granules throughout; preapical stripe of elytron reduced, shorter than one-fifth of elytral length	***Pseudoechthistatus granulatus***
–	Elytra comparatively short, 1.8 times (in males) or 2.0 times (in females) as long as humeral width; elytral disk sparsely covered with moderately sized granules which become indistinct near apical one-third, and absent beyond apical one-fourth; preapical stripe of elytron developed, subequal to one-fourth of elytral length	***Pseudoechthistatus sinicus* sp. n.**
6	Elytron distinctly granulated, granules reaching at least apical one-fourth; punctures sparse and shallow	**7**
–	Elytron weakly granulated, granules reaching at most basal one-third; punctures distinct and deep, at least reaching middle	**8**
7	Elytral granules smaller and dense; middle band narrow, moderately oblique, interrupted or nearly so at middle	***Pseudoechthistatus chiangshunani* sp. n.**
–	Elytral granules larger and sparse; middle band broad, nearly transverse, not interrupted or narrowed	***Pseudoechthistatus birmanicus***
8	Elytral punctures moderate, reaching the middle; at least 3^rd^ to 4^th^ antennomeres annulate with light pubescence at base; elytral middle band interrupted or nearly so at middle	***Pseudoechthistatus holzschuhi* sp. n.**
–	Elytral punctures deep and coarse, reaching the apex; antennomeres without distinct annular light pubescence; elytral middle band complete	***Pseudoechthistatus pufujiae* sp. n.**

## Supplementary Material

XML Treatment for Pseudoechthistatus

XML Treatment for Pseudoechthistatus obliquefasciatus

XML Treatment for Pseudoechthistatus acutipennis

XML Treatment for Pseudoechthistatus birmanicus

XML Treatment for Pseudoechthistatus granulatus

XML Treatment for Pseudoechthistatus sinicus

XML Treatment for Pseudoechthistatus chiangshunani

XML Treatment for Pseudoechthistatus holzschuhi

XML Treatment for Pseudoechthistatus pufujiae

XML Treatment for Pseudoechthistatus glabripennis
